# Uniform transgene activation in Tet-On systems depends on sustained rtTA expression

**DOI:** 10.1016/j.isci.2023.107685

**Published:** 2023-08-19

**Authors:** Jun Otomo, Knut Woltjen, Hidetoshi Sakurai

**Affiliations:** 1 Center for iPS Cell Research and Application (CiRA), Kyoto University, Sakyo-ku, Kyoto 606-8507, Japan

**Keywords:** Cell biology, Stem cells research, Methodology in biological sciences

## Abstract

Application of the tetracycline-inducible gene expression system (Tet-On) in human induced pluripotent stem cells (hiPSCs) has become a fundamental transgenic tool owing to its regulatable gene expression. One of the major hurdles in hiPSC application is non-uniform transgene activation. Here, we report that the supplementation of reverse tetracycline transactivator (rtTA) in polyclonal hiPSCs populations can achieve the uniform transgene activation of Tet-On. Furthermore, the choice of antibiotic selection markers connected by an internal ribosomal entry site (IRES) can influence the expression of upstream transgenes. In particular, expression of the rtTA is more uniform in cell populations when linked to puromycin as compared to neomycin, obviating the need for sub-cloning or supplementation of rtTA. Finally, to expand the range of applications, we adopted our findings to tetracycline-inducible MyoD vector (Tet-MyoD). Our Tet-MyoD promises efficient, robust, and reproducible directed myogenic differentiation of hiPSCs.

## Highlights

• Expression of rtTA is unstable in hiPSCs when linked to neomycin resistance gene
• Uniform transgene activation of Tet-On can be achieved by rtTA supplementation
• Expression of rtTA sustains in hiPSCs when linked to puromycin resistance gene
• Sustained rtTA expression in hiPSCs achieves uniform myogenic differentiation

## Graphical abstract

**Figure:**
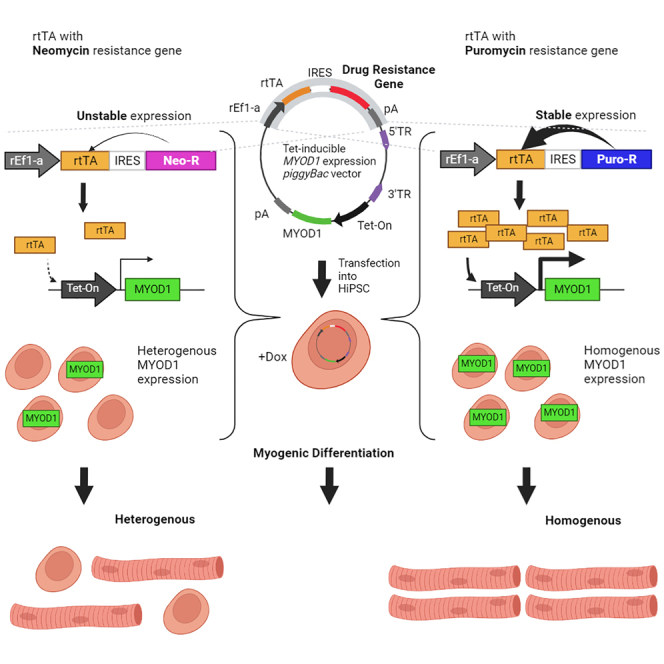

## Introduction

The tetracycline-responsive regulatory system^1^ has become a fundamental genetic tool owing to its easily regulatable transgene expression. One of the major reasons for its widespread appeal is the efficient transgene expression driven by the tetracycline-controlled gene expression (Tet-On) promoter in response to doxycycline (dox). The Tet-On promoter consists of the minimal cytomegalovirus (CMV) promoter fused to repeats of the Tet-operon sequence from *Escherichia coli*.^2^ A key component of the Tet-On system is the reverse tetracycline transactivator (rtTA), a fusion of a mutant tetracycline repressor and the activation domain of the herpes simplex virus virion protein 16 (VP16).^2^ Once dox is added to cells with the Tet-On system, rtTA binds to Tet-operon repeats and activates transgene expression from the minimal CMV promoter.^2^ Since its establishment, the Tet-On system has been employed to investigate specific gene functions in mammalian cells and the direct differentiation of human induced pluripotent stem cells (hiPSCs).

The establishment of hiPSCs and differentiation into specific cell lineages have allowed the creation of new *in vitro* disease models.^3^^,^^4^ A common approach for differentiating hiPSCs is the forced expression of a lineage-specific master transcription factor. The Tet-On system plays an important role in this strategy as it allows researchers to begin the direct differentiation of several cell lineages from hiPSCs at predefined times.^5^^,^^6^^,^^7^ The *piggyBac* transposon is a non-viral gene delivery system that achieves efficient and stable genomic integration by transposition in mammalian cells.^8^^,^^9^ Its large cargo capacity^10^ provides an advantage over viral vectors by allowing the introduction of complex transgenic constructs such as the Tet-On system in a single vector.^11^ However, one of the major hurdles in hiPSCs applications is non-uniform transgene activation. Therefore, clonal evaluation and selection are frequently needed to identify cell lines that express the transgene efficiently and stably.

Previously, we described the directed differentiation of hiPSCs into skeletal muscle cells by forced expression of myogenic differentiation 1 (*MYOD1*)^5^^,^^12^ which encodes MyoD, a master transcription factor of myogenic cells.^13^ Expressing *MYOD1*^13^ under the Tet-On system (Tet-MyoD) delivered by *piggyBac* allowed us to differentiate hiPSC clones into myocytes with high efficiency and reproducibility within just 7 days by simply adding dox to the culture medium. Furthermore, application of this approach to patient-derived hiPSCs improved our understanding of several types of muscular diseases.^14^^,^^15^^,^^16^^,^^17^ We also adapted our method for high-throughput screening, with which we identified a possible candidate drug for Miyoshi myopathy.^18^ One major limitation has been inconsistent transgene activation in some populations of Tet-MyoD hiPSCs such that after transposition it was necessary to select clones that expressed *MYOD1* uniformly and could efficiently differentiate into skeletal muscle cells.^5^^,^^12^ Still, it would be beneficial to use polyclonal populations directly as it saves time and resources and prevents potential bias during clonal selection. Thus, improving the consistency of transgene activation remains a challenge that, once overcome, would improve the range of applications*.* However, cause of non-uniform transgene activation and possible solutions remain to be elucidated.

Recently, Duran et al. have shown that a lower level of rtTA expression is one of the major causes for non-uniform transgene activation.^19^ Here we demonstrated that reduced expression level of rtTA was observed in cells without transgene activation, whereas the supplementation of rtTA expression sufficiently improved both transgene activation and myogenic differentiation in the populations. However, supplementation of rtTA expression is often complicated as it requires additional transgene expressing vector to be introduced in the cells. Therefore, new solutions to sustain rtTA expression without any need for its supplementation are highly demanded. It has been suggested that transgene expression may be affected by antibiotic selection markers. Selection with puromycin, rather than with G418, led to higher reporter gene expression in mouse embryonic stem cells (mESCs)^20^ and human embryonic stem cells (hESCs).^21^ Furthermore, non-uniform transgene activation of Tet-On system has been observed in both G418-selected mESCs^22^ and hiPSC clones in our previous study.^5^ These suggest that using puromycin as a selection marker could be a possible solution for non-uniform transgene activation.

Here, we have employed all-in-one *piggyBac* tetracycline-inducible transgene vectors which constitutively express rtTA with either neomycin resistance gene or puromycin resistance gene. We have demonstrated that conjugating the puromycin resistance gene to rtTA with internal ribosomal entry site (IRES) sequence in Tet-On system sustained its expression and improved both uniform transgene expression and myogenic differentiation without the need for sub-cloning or rtTA supplementation.

Our findings are expected for future application of hiPSCs research including studying specific transgene function and directed differentiation of specific cell lineage any other than myogenic cells as previously reported.^6^^,^^7^

## Results

### Failure of transgene activation in Tet-MyoD hiPSCs populations associated with reduced rtTA expression

**Figure 1:**
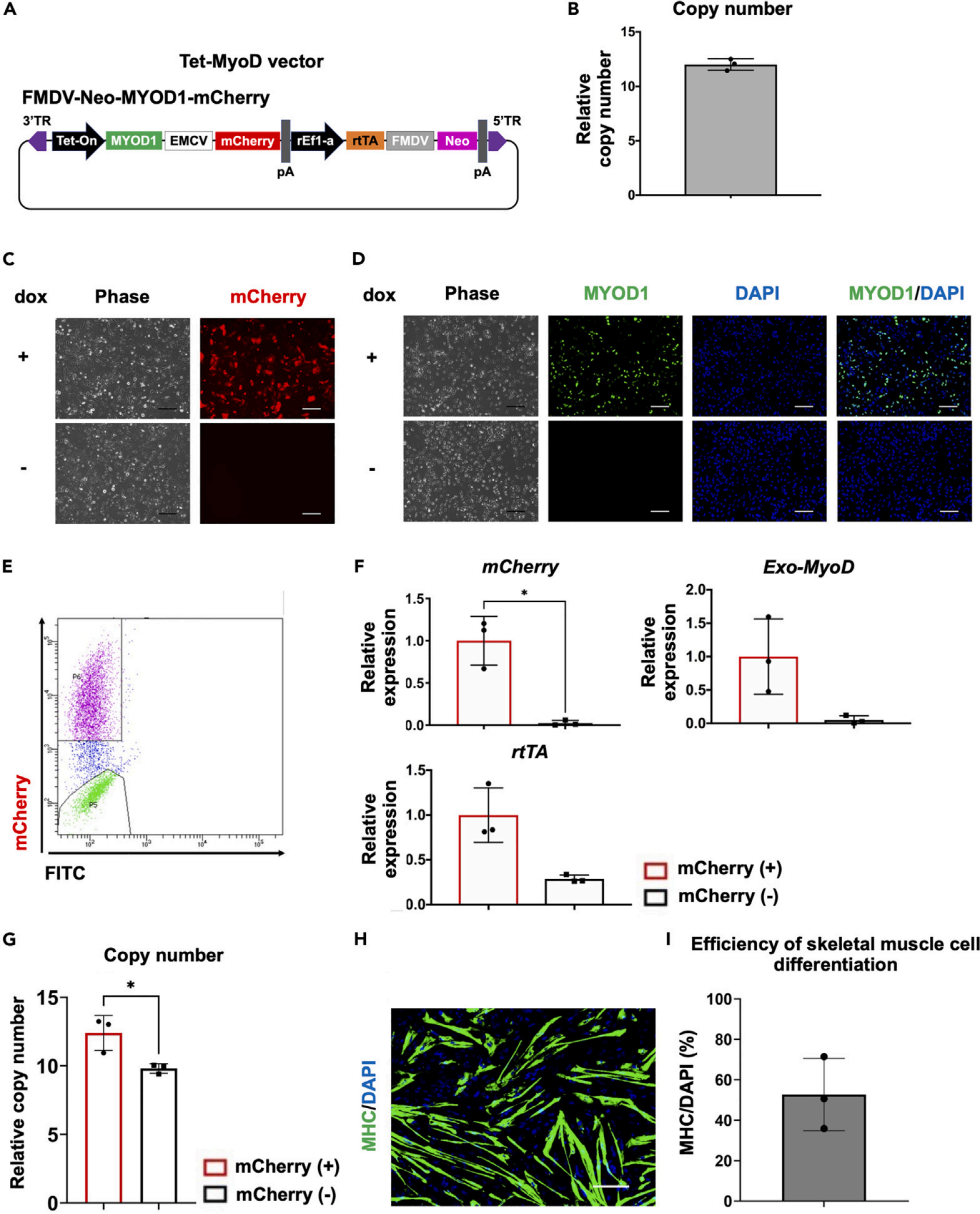
Evaluating transgene expression and myogenic differentiation efficiency in the total population of Tet-MyoD hiPSCs (A) Diagram of All-in-One vector designed to express dox-inducible *MYOD1* (Tet-MyoD) with co-expression of the mCherry reporter gene. Vector components include a Tet-On dox-inducible promoter, internal ribosome entry site from foot-and-mouth disease virus (FMDV) or encephalomyocarditis virus (EMCV), polyadenylation signal (pA), rat elongation factor 1a (rEF1a) promoter, reverse tetracycline transactivator (rtTA), and neomycin phosphotransferase (*Neo*). (B) Quantitative PCR (qPCR) analysis for Tet-MyoD copy number integrations in generated FMDV-Neo-MYOD1-mCherry populations. The rtTA primer was used for detecting Tet-MyoD vectors, and DLX5 was used as an internal control. A single copy number-integrated Tet-MyoD vector cell line was used as the reference. Values represent means ± SD, n = 3. (C) Phase-contrast and fluorescence images showing mCherry expression in the generated Tet-MyoD hiPSCs. Images were taken after 24 h of dox treatment (top row) or without dox (bottom row). Scale bars, 200 μm. (D) Phase-contrast and fluorescence images showing immunostaining of the myogenic differentiation marker MYOD1. DAPI was used for counter-staining. Cells were fixed and stained after 24 h of dox treatment (top row) or without dox (bottom row). Scale bars, 200 μm. (E) mCherry fluorescence-activated cell sorting (FACS) of FMDV-Neo-MYOD1-mCherry populations after 24 h of 1.5 μg/mL dox treatment. Representative graphs are shown. (F) qPCR results showing *mCherry*, *Exogenous MyoD* (*Exo-MyoD*), and *rtTA* expressions in mCherry-positive and mCherry-negative populations sorted from FMDV-Neo-MYOD1-mCherry populations. Relative expression levels were normalized to *PBGD* as an internal control in each sample and then to mCherry-positive populations. Values represent means ± SD, n = 3. (G) qPCR analysis for Tet-MyoD copy number integrations in mCherry-positive and mCherry-negative populations sorted from FMDV-Neo-MYOD1-mCherry populations. The rtTA primer was used for detecting Tet-MyoD vectors, and DLX5 was used as an internal control. A single copy number-integrated Tet-MyoD vector cell line was used as the reference. Values represent means ± SD, n = 3. (H) Immunostaining for the skeletal muscle cell marker myosin heavy chain (MHC) (green). DAPI (blue) was used for counter-staining. Differentiated cells were fixed and stained on d7. Scale bars, 200 μm. (I) Quantification of skeletal muscle cell differentiation efficiency of the Tet-MyoD hiPSCs. Efficiency was calculated by counting the total number of nuclei (blue) in MHC-positive cells (green) and then dividing the number by the total number of nuclei. Values represent means ± SD, n = 3. ∗p < 0.05 according to Paired *t* test.

Using all-in-one *piggyBac* tetracycline-inducible MyoD vector, overexpressing MYOD1 in hiPSCs can directly differentiate into skeletal muscle cells.^5^ The vector, FMDV-Neo-MYOD1-mCherry, was designed to drive MYOD1 with mCherry under dox-responsive promoter and rtTA under a rat elongation factor 1a (rEF1a) promoter, which was conjugated to the neomycin resistance gene with an IRES sequence from the foot-and-mouth disease virus (FMDV)^5^ (Figure 1A). As previously shown, FMDV-Neo-MYOD1-mCherry vector has a dox dose dependency for its tetracycline-inducible transgene expression (Figure S1A).^11^ The vector was electroporated into hiPSCs line Healthy Control #1 (HC #1) along with a *piggyBac* transposase expression vector and followed by the scheme (Figure S1B). After antibiotic G418 selection and 3 passages from the electroporation (PE 3), Tet-MyoD hiPSCs populations FMDV-Neo-MYOD1-mCherry was generated with multiple copy number integrations of Tet-MyoD vector (Figure 1B). The populations were subjected to myogenic differentiation as described previously (Figure S1C)^12^ and subsequent analysis. During the myogenic differentiation of FMDV-Neo-MYOD1-mCherry populations, we observed both mCherry-positive and mCherry-negative populations after 24 h of dox treatment (Figure 1C) with similar outcome of MYOD1 expression (Figure 1D). Furthermore, as most of mCherry-positive populations appeared with positive for pan-myosin heavy chain (MHC), a marker of skeletal muscle cell differentiation, after myogenic differentiation (Figures S1D and S1E), it has been suggested that improving the mCherry/MYOD1-positive populations may improve the myogenic differentiation. To explore the cause of mCherry positive and negative cells in the populations, we separated these cells by fluorescence-activated cell sorting (FACS) (Figure 1E) and subjected them to subsequent analysis of the transgene expression and Tet-MyoD vector copy number integrations. The expressions of *mCherry* and *Exo-MyoD* were higher in mCherry-positive populations than those in mCherry-negative populations (Figure 1F) as expected. Interestingly, *rtTA* expression was significantly lower in mCherry-negative populations which was more than 2 times lower in the populations even though that was driven by the constitutive active rEF1a promoter (Figure 1F), while that of Tet-MyoD vector copy number did not show such large differences (Figure 1G).

On the last day of differentiation (d7), cells appeared as both positive and negative for MHC (Figure 1H). Quantitative image analysis revealed more than 20% of the cells were failed in myogenic differentiation (Figure 1I).

After several weeks or passages (PE 8 and 12), comparing to the beginning of establishment (PE 3), increased copy number integrations of Tet-MyoD vectors in the populations were observed (Figures 1B and S1F). Although the populations improved their copy number integrations, flow cytometry (FCM) analysis revealed around 20% of the populations were negative for mCherry expression (Figure S1G). Moreover, after separating mCherry-positive and mCherry-negative populations by FACS, it has been revealed that *rtTA* expression was still significantly lower in mCherry-negative populations (Figure S1H) even though they showed multiple copy number integrations (Figure S1I). Especially at PE 12 (after 12 passages from the electroporation), *rtTA* expression level was significantly lower in mCherry-negative cells (Figure S1H) without showing significant difference of copy number integrations (Figure S1I). At the end of the differentiation, the populations appeared as both positive and negative for MHC (Figure S1J). These data indicated that non-uniform transgene activation and different rtTA expression levels in the populations were independent of their copy number integrations.

### rtTA supplementation sufficiently improves both transgene activation and myogenic differentiation

It has been suggested that reduction of rtTA expression is one of the major causes of non-uniform transgene activation of Tet-On and the supplementation of rtTA improves the transgene activation.^19^ To investigate whether rtTA expression affects both transgene activation and myogenic differentiation in Tet-MyoD hiPSCs, we attempted to rescue rtTA expression by simultaneously transfecting FMDV-Neo-MYOD1-mCherry vector with an additional CMV enhancer/chicken b-actin (CAG) promoter driving rtTA (CAG-rtTA-EGFP) vector.^23^ In the CAG-rtTA-EGFP vector, rtTA is conjugated to EGFP through an encephalomyocarditis virus (EMCV) IRES and its expression is driven by the constitutive CAG promoter.^24^

**Figure 2:**
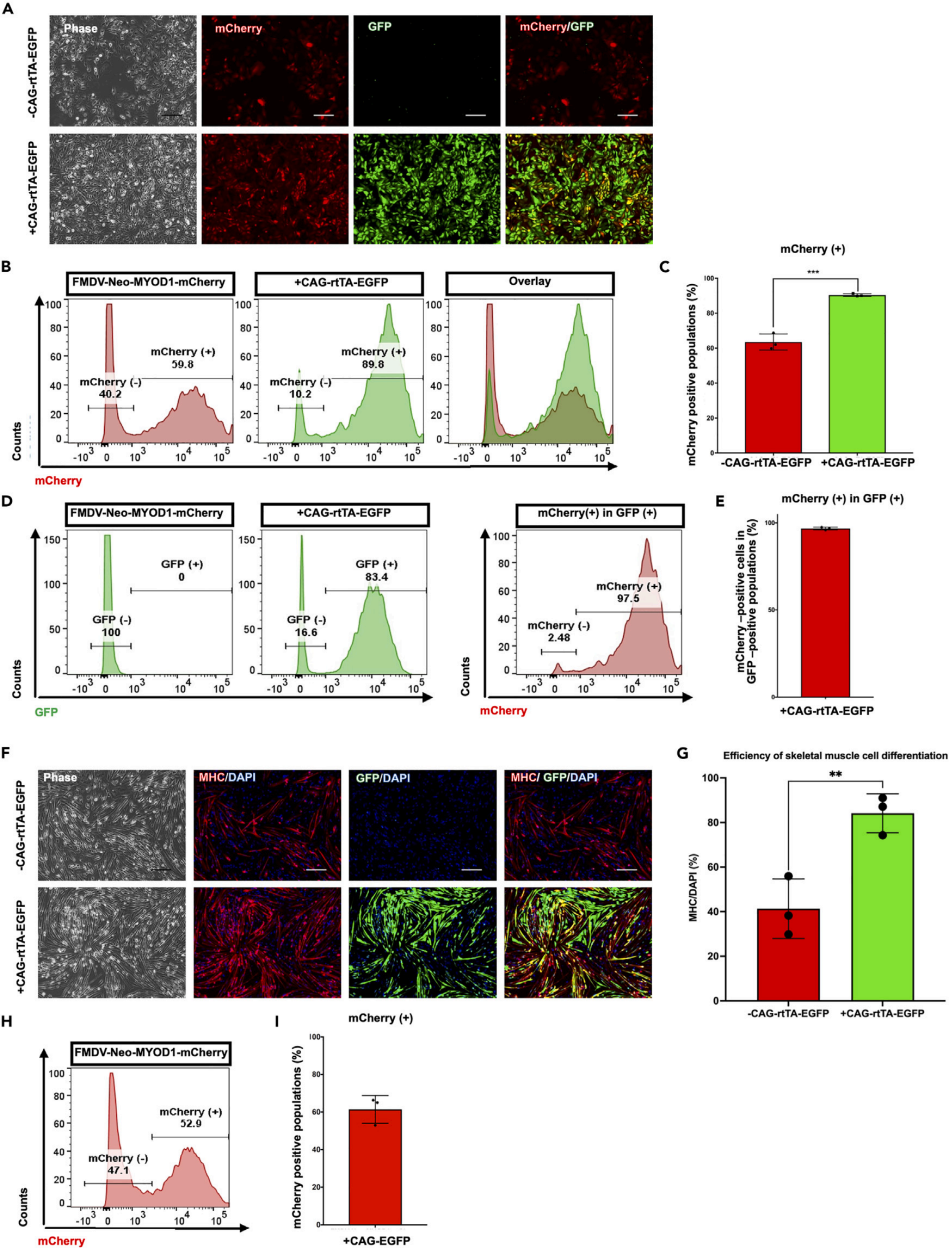
Additional expression of rtTA improves tetracycline-inducible mCherry expression and efficiency of myogenic differentiation (A) Phase-contrast and fluorescence images showing mCherry and GFP expression in the generated FMDV-Neo-MYOD1-mCherry populations with (bottom row) or without (top row) CAG-rtTA-EGFP. After the generation of populations, the cells were reseeded on Matrigel-coated plates, and 48 h later 1.5 μg/mL dox was added to the culture medium. Images were taken 24 h after dox treatment. Scale bars, 200 μm. (B) mCherry flow cytometry (FCM) analysis in FMDV-Neo-MYOD1-mCherry populations with (middle panel) or without (left panel) CAG-rtTA-EGFP. Right panel shows overlayed data. Representative graphs are shown. (C) Quantification of mCherry-positive populations from the FCM data in Figure 2B. Values represent means ± SD, n = 3. (D) GFP FCM analysis in FMDV-Neo-MYOD1-mCherry populations with (right panel) or without (left panel) CAG-rtTA-EGFP. The mCherry FCM analysis of GFP-positive populations in the same cells is also shown. Representative graphs are shown. (E) Quantification of the mCherry-positive percentage in the GFP-positive populations from Figure 2D. Values represent means ± SD, n = 3. (F) Immunostaining for the skeletal muscle cell marker MHC (red) and GFP fluorescence (green) images. DAPI (blue) was used for counter-staining. Differentiated cells were fixed and stained on day 7 of the differentiation. Scale bars, 200 μm. (G) Quantification of skeletal muscle cell differentiation efficiency from Figure 2F. Values represent means ± SD, n = 3. (H) mCherry FCM analysis in FMDV-Neo-MYOD1-mCherry populations with CAG-EGFP. Representative graph is shown. (I) Quantification of mCherry-positive populations from the FCM data in Figure 2H. Values represent means ± SD, n = 3. ∗∗p < 0.01,∗∗∗p < 0.001 according to Student’s *t* test (unpaired).

After generating Tet-MyoD hiPSCs with CAG-rtTA-EGFP, these cells are subject to myogenic differentiation. After 24 h of dox treatment, we observed that cells expressing GFP co-localized with mCherry (Figure 2A). FCM analysis revealed that co-transfection with CAG-rtTA-EGFP increased significantly the number of mCherry-positive cells in the populations (Figures 2B and 2C). Gating on the GFP-positive population, nearly 100% of the GFP-positive population was positive for mCherry (Figures 2D and 2E). Importantly, after continuous culture of these cells up to d7, we also observed improved myogenic differentiation in additional rtTA-expressing cells (Figures 2F and 2G), and most GFP-positive cells were pan-MHC positive (Figure 2F). An EGFP-expressing vector (CAG-EGFP)^25^ was also co-transfected into hiPSCs as a negative control; it did not affect the rates of mCherry populations as they were similar to those of single transfecting FMDV-Neo-MYOD1-mCherry (Figures 2H and 2I). Overall, a lower rtTA expression appears to be a major cause of Tet-On promoter dysfunction, and supplementation of its expression sufficiently improves both transgene activation and myogenic differentiation in the populations.

### Different *rtTA* expression levels observed between neomycin and puromycin-selected populations

**Figure 3:**
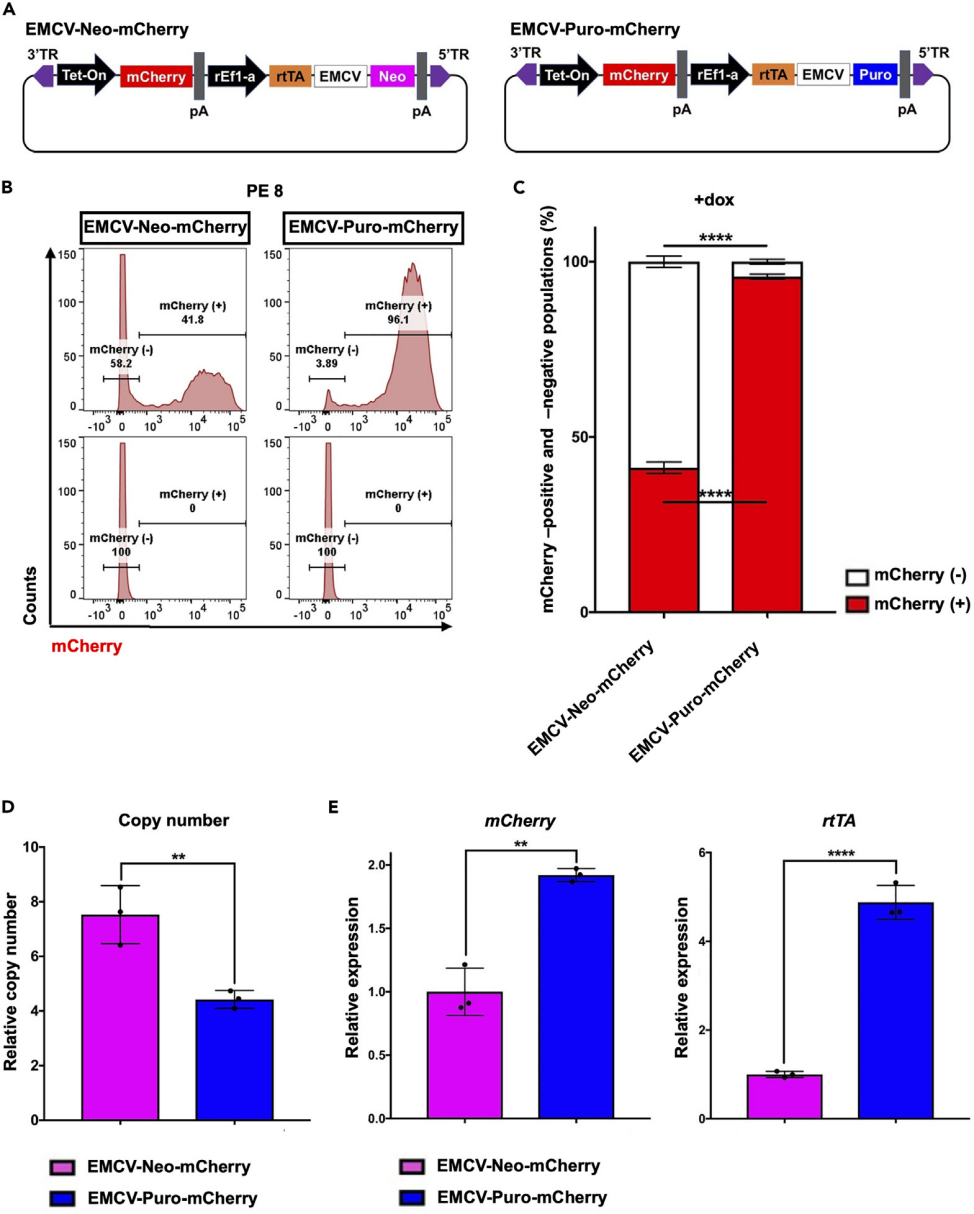
Evaluating transgene expression in Tet-mCherry hiPSCs after the extended culture (A) Diagram of two different All-in-One vectors designed to express dox-inducible *mCherry* (Tet-mCherry). EMCV-Neo-mCherry vector contains neomycin phosphotransferase (*Neo*), and EMCV-Puro-mCherry contains puromycin N-acetyltransferase (Puro). (B) mCherry FCM analysis of EMCV-Neo-mCherry and EMCV-Puro-mCherry populations at PE 8, after 24 h of 1.5 μg/mL dox treatment (top row) or without dox (bottom row). Representative images are shown. (C) Quantification of mCherry-positive (red) and mCherry-negative populations (white) from the FCM data in Figure 3B. (D) qPCR analysis for Tet-mCherry copy number integrations in the generated Tet-mCherry hiPSCs. Values represent means ± SD, n = 3. (E) qPCR results showing *mCherry*, and *rtTA* expressions in EMCV-Neo-mCherry and EMCV-Puro-mCherry populations 24 h after 1.5 μg/mL dox treatment. Relative expression levels were normalized to *PBGD* as an internal control in each sample and then to EMCV-Neo-mCherry populations. Values represent means ± SD, n = 3. ∗∗p < 0.01, ∗∗∗∗p < 0.0001 according to Student’s *t* test (unpaired).

It has been suggested that transgene expression may be affected by antibiotic resistance markers^20^^,^^21^ or choice of IRES for co-expression.^26^^,^^27^^,^^28^^,^^29^^,^^30^ To investigate the effect of these factors on Tet-On system, we generated two different customized *piggyBac* tetracycline-inducible mCherry expression vectors, with the same construct except for their antibiotic resistance gene (Figure 3A), based on a vector from previous study.^11^ The vectors contained a Tet-On promoter driving mCherry and a rEF1a constitutive promoter driving rtTA, which was conjugated to the neomycin (EMCV-Neo-mCherry) or puromycin resistance gene (EMCV-Puro-mCherry) with an IRES sequence form the EMCV (Figure 3A).^31^^,^^32^ The vectors were electroporated separately into hiPSCs line HC #1 along with a *piggyBac* transposase expression vector. After 48 h, the cells were selected with either G418 or puromycin, cultured under selection pressure (Figure S2A), and subjected to subsequent analysis with several passages (Figures S2A and S2B). As similar results in Figure 1, established populations showed multiple copy number integrations of the Tet-mCherry vectors (Figure S2C), and 1 day after dox treatment, both mCherry-positive and mCherry-negative cells were observed in EMCV-Neo-mCherry populations whereas EMCV-Puro-mCherry showed more uniformed populations of mCherry-positive cells (Figure S2D). FCM analysis showed less than 60% of the populations were positive for mCherry in EMCV-Neo-mCherry and more than 90% of the populations were positive in EMCV-Puro-mCherry at both beginning of the establishment PE 3 (Figures S2E and S2F) and late PE 8 (Figures 3B and 3C). Despite lower copy number integrations in EMCV-Puro-mCherry populations comparing to those of EMCV-Neo-mCherry populations, they showed higher transgene expression of both *mCherry* and *rtTA* (Figures 3D and 3E). Importantly, copy number of integrations had been increased in EMCV-Neo-mCherry populations (Figure 3D) from beginning of the establishment (Figure S2C), suggesting cells without vector integration no longer remained in the populations and thus there should be no leaky selection with G418. Overall, these data suggested EMCV-Puro-mCherry populations permit both higher *rtTA* expression and uniform transgene activation of Tet-On promoter without showing any advantages of copy number integrations.

### Failed transgene activation associated with lower *rtTA* expression level but it is unlikely copy number-dependent manner

**Figure 4:**
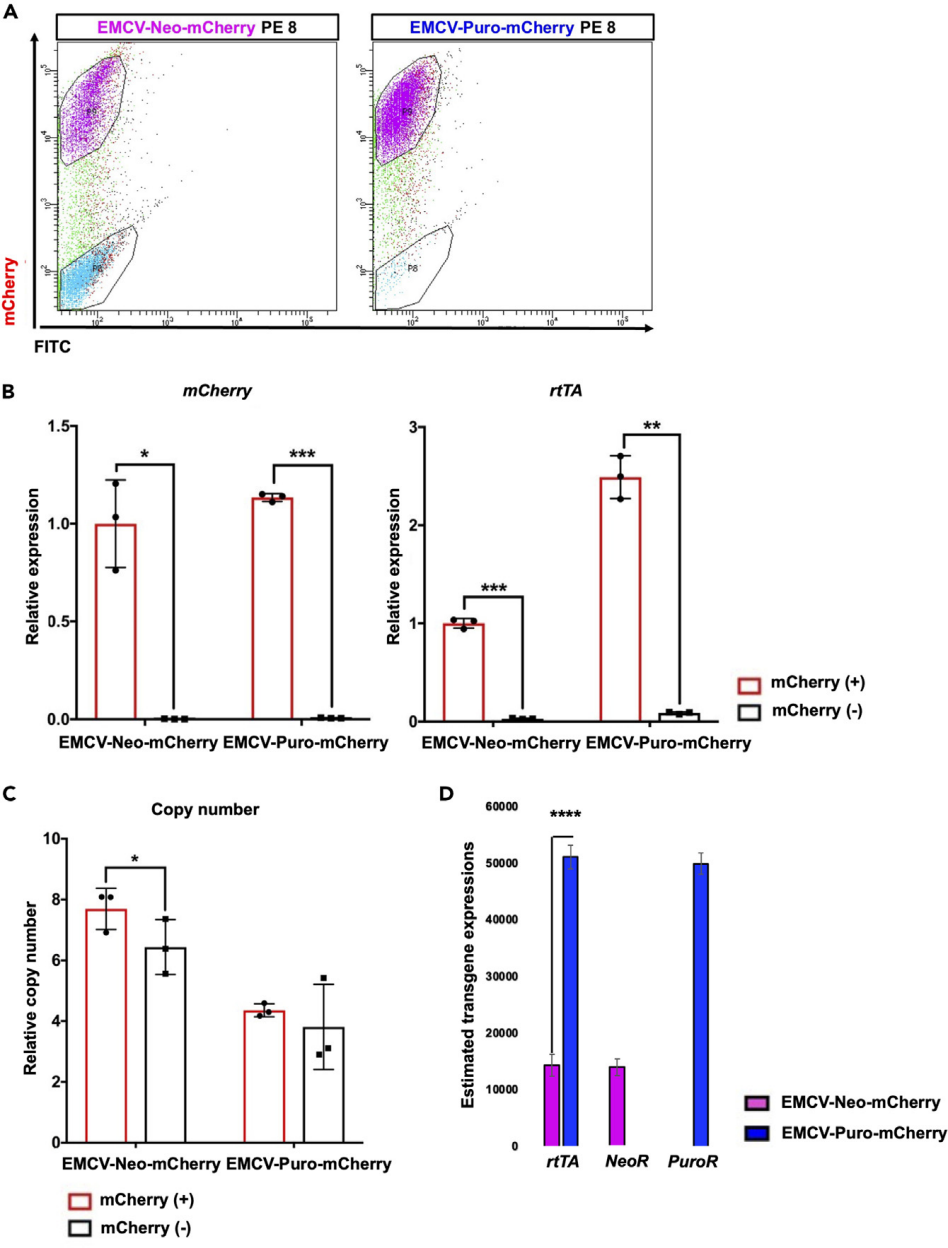
Comparing transgene expressions between mCherry-positive and mCherry-negative populations in Tet-mCherry hiPSCs (A) mCherry FACS of Tet-mCherry hiPSCs after 24 h of 1.5 μg/mL dox treatment. Representative graphs are shown. (B) qPCR results showing *mCherry* and *rtTA* expressions in mCherry-positive and mCherry-negative populations sorted from EMCV-Neo-mCherry and EMCV-Puro-mCherry populations. Relative expression levels were normalized to *PBGD* as an internal control in each sample and then to mCherry-positive populations of EMCV-Neo-mCherry. Values represent means ± SD, n = 3. (C) qPCR analysis for Tet-mCherry copy number integrations in mCherry-positive and mCherry-negative populations sorted from Tet-mCherry hiPSCs. The rtTA primer was used for detecting Tet-mCherry and Tet-MyoD vectors, and DLX5 was used as an internal control. A single copy number-integrated Tet-MyoD vector cell line was used as the reference. Values represent means ± SD, n = 3. (D) qPCR results showing *rtTA, NeoR*, *and PuroR* expressions in mCherry-positive populations sorted from EMCV-Neo-mCherry and EMCV-Puro-mCherry populations. qPCR for *NeoR* was performed only in EMCV-Neo-mCherry populations and qPCR for *PuroR* was performeed only in EMCV-Puro-mCherry populations. A standard curve was constructed for each transgene by serially diluting the plasmid vector. Data were normalized to the amount of total RNA (15 ng) used for the cDNA synthesis. Values represent means ± SD, n = 3. ∗p < 0.05 ∗∗p < 0.01, ∗∗∗p < 0.001, ∗∗∗∗p < 0.0001 according to Paired *t* test in (B) and (C) and Student’s *t* test (unpaired) in (D).

For the further analysis, we separated the positive and negative mCherry cells by FACS for subsequent analysis of the transgene expression and Tet-mCherry vector copy number (Figure 4A). The expressions of *mCherry* and *rtTA* were higher in mCherry-positive populations (Figure 4B) as observed in myogenic differentiation (Figure 1). Despite both mCherry-positive and mCherry-negative cells showing multiple copy number integrations in EMCV-Neo/Puro-mCherry populations (Figure 4C), *rtTA* expression level is dramatically downregulated in mCherry-negative populations (Figure 4B). Moreover, comparing to both mCherry-positive and mCherry-negative cells in EMCV-Neo-mCherry, EMCV-Puro-mCherry with mCherry-positive cells tend to be lower in copy number of integrations (Figure 4C), but they showed higher expression of *rtTA* (Figure 4B). One of the major considerations encountered in bicistronic transgene expression system with IRES is different transgene expression level between primary and secondary transgene on the same mRNA.^33^ This is explained by different translation efficiency. CAP-dependent translation is more efficient than IRES-dependent translation; thus, primary transgene shows higher protein expression than secondary transgene.^33^ For transcription, it can be predicted that both primary and secondary transgene are expressed at similar efficiency as they are transcribed into one fusion mRNA with IRES sequence. As we predicted, using plasmid vectors as a standard control in qPCR analysis and based on their calculated copy number, it has been revealed that *rtTA* has similar transgene copy number to that of *Puromycin resistance gene* (*PuroR*) and *Neomycin resistance gene* (*NeoR)* in mCherry-positive populations (Figure 4D), which could be explained by the fact that both transgenes are transcribed as a fusion mRNA with IRES (Figure S3A). Interestingly, both primary transgene of rtTA and secondary transgene of antibiotic resistance gene show higher transgene copy number in EMCV-Puro-mChery (Figure 4D) and *rtTA* expression is significantly higher (Figures 4B and 4D). Transgene copy number for *mCherry* shows the largest value (Figure S3B), suggesting Tet-On promoter with the highest activity in the construct. Overall, through the results of Tet-MyoD and Tet-mCherry, it has been revealed that *rtTA* expression level is a major responsibility for activating Tet-On system as previously reported^19^ and puromycin-selected populations sustain their rtTA expression to achieves uniform transgene activation without showing advantages of copy number integrations. Moreover, fusion mRNA of *rtTA* and *PuroR* can be more efficiently transcribed than fusion mRNA of *rtTA* and *NeoR* in the populations.

### Tet-MyoD equipping puromycin resistance gene permits uniform transgene activation and higher myogenic differentiation efficiency in hiPSCs

**Figure 5:**
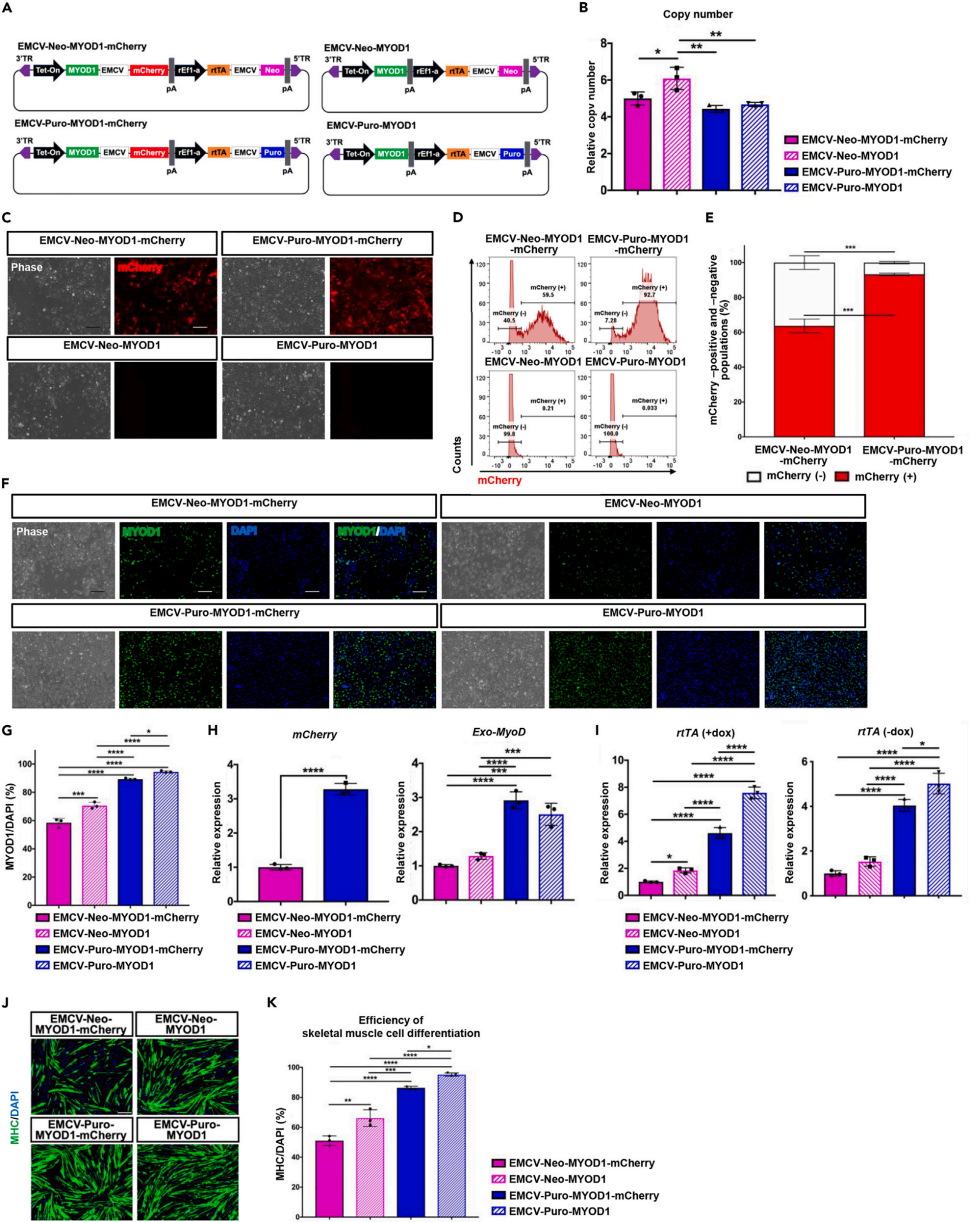
Generation of Neomycin-selected Tet-MyoD hiPSCs and Puromycin-selected Tet-MyoD hiPSCs (A) Diagram of four different All-in-One vectors designed to express dox-inducible *MYOD1* (Tet-MyoD) with (left) or without (right) co-expression of the mCherry reporter gene. (B) qPCR analysis for Tet-MyoD copy number integrations in generated Tet-MyoD hiPSCs. Values represent means ± SD, n = 3. (C) Phase-contrast and fluorescence images showing mCherry expression in the generated Tet-MyoD hiPSCs. Images were taken after 24 h of dox treatment. Scale bars, 200 μm. (D) mCherry FCM analysis of Tet-MyoD hiPSCs after 24 h of 1.5 μg/mL dox treatment. Representative graphs are shown. (E) Quantification of mCherry-positive (red) and mCherry-negative populations (white) from the FCM data in Figure 5D. Values represent means ± SD, n = 3. (F) Phase-contrast and fluorescence images showing immunostaining of the myogenic differentiation marker MYOD1. DAPI was used for counter-staining. Cells were fixed and stained after 24 h of dox treatment. (G) Quantification of MYOD1-positive populations of the Tet-MyoD hiPSCs. MYOD1-positive ratio was calculated by counting the total number of nuclei (blue) in MYOD1-positive cells (green) and then dividing the number by the total number of nuclei. Values represent means ± SD, n = 3. (H and I) qPCR analysis showing *mCherry*, *Exo-MyoD* and (I) *rtTA* expression in Tet-MyoD hiPSCs 24 h after 1.5 μg/mL dox treatment or without dox (in the case of rtTA only). Relative expression levels were normalized to *PBGD* as an internal control in each sample and then to EMCV-Neo-MYOD1-mCherry populations. Values represent means ± SD, n = 3. (J) Immunostaining for the skeletal muscle cell marker MHC (green). DAPI (blue) was used for counter-staining. Differentiated cells were fixed and stained on d7. Scale bars, 200 μm. (K) Quantification of skeletal muscle cell differentiation efficiency of the Tet-MyoD hiPSCs. Values represent means ± SD, n = 3. ∗p < 0.05, ∗∗p < 0.01, ∗∗∗p < 0.001, ∗∗∗∗p < 0.0001 according to one-way ANOVA followed by Tukey’s test in (B), (G), (H), (I), and (K) and Student’s *t* test (unpaired) in (E) and (H).

To expand the range of applications, we adopted our findings to Tet-MyoD vector. We generated four different customized *piggyBac* Tet-MyoD expression vectors based on a vector from our previous study^5^ (Figure 1A). The vector series all contained a dox-responsive Tet-On promoter driving *MYOD1* expression with or without *mCherry* and a rat elongation factor 1a (rEF1a) constitutive promoter driving *rtTA*, which was conjugated to the *neomycin* or *puromycin* resistance genes with an IRES sequence of EMCV. The vectors were named EMCV-Neo-MYOD1, EMCV-Neo-MYOD1-mCherry, EMCV-Puro-MYOD1,^12^ and EMCV-Puro-MYOD1-mCherry (Figure 5A). These four different vectors were electroporated separately into hiPSCs line HC #1 along with a *piggyBac* transposase expression vector and followed by the scheme previously described (Figures S1B and S2A). After antibiotic selection, we generated four polyclonal Tet-MyoD hiPSC populations, which were named according to the electroporated Tet-MyoD vectors. According to copy number analysis, puromycin-selected populations, EMCV-Puro-MYOD1 and EMCV-Puro-MYOD1-mCherry, showed lower copy number of Tet-MyoD vector, comparing to that of neomycin-selected populations, EMCV-Neo-MYOD1 and EMCV-Neo-MYOD1-mCherry (Figure 5B). However, after the populations were subjected to myogenic differentiation and subsequent analysis, both mCherry-positive and mCherry-negative cells were observed in EMCV-Neo-MYOD1-mCherry populations (Figures 5C and 5D). It has been revealed that nearly 90% of EMCV-Puro-MYOD1-mCherry populations were positive for mCherry expression, whereas 60% of EMCV-Neo-MYOD1-mCherry populations were positive after 24 h of dox treatment (Figures 5D and 5E). Similarly, while 60%–70% of EMCV-Neo-MYOD1/-mCherry populations were positive for MYOD1 expression, more than 80% of EMCV-Puro-MYOD1/-mCherry populations were MYOD1 positive (Figures 5F and G). As we expected, both *mCherry* and *Exo-MyoD* were highly expressed in EMCV-Puro-MYOD1/-mCherry populations comparing to those of EMCV-Neo-MYOD1/-mCherry populations (Figure 5H). Notably, *rtTA* was also highly expressed in EMCV-Puro-MYOD1/-mCherry with or without dox (Figure 5I). On the last day of myogenic differentiation, EMCV-Puro-MYOD1/-mCherry populations achieved more than 80% of skeletal muscle cells differentiation efficiency, whereas neomycin-selected populations showed less than 80% efficiency (Figures 5J and 5K). Interestingly, while puromycin-selected populations showed uniform transgene activation and myogenic differentiation, those improvements were also observed between with and without mCherry cassette in Tet-MyoD vector, EMCV-Neo-MYOD1 and EMCV-Neo-MYOD1-mCherry or EMCV-Puro-MYOD1 and EMCV-Puro-MYOD1-mCherry (Figure 5F, G, J, and K). This improvement could be explained by the fact that populations with monocistronic expression vectors are preferred to translate MYOD1 without considering any problems encountered in bicistronic expression vectors as previously reported.^28^^,^^34^

Among several passages of Tet-MyoD hiPSCs (PE 3,8, and 12), as we observed previously (Figure S1F), copy number of Tet-MyoD vector integrations in both EMCV-Neo-MYOD1/-mCherry populations had been slightly increased (Figure S4A), and thus there should be no leaky selection of G418. Despite increased copy number integrations in neomycin-selected populations during several passages, both mCherry-positive populations and efficiency of myogenic differentiation remained to be lower in the populations while those of puromycin-selected populations remained to be higher (Figures S4B and S4C) without affecting their karyotype or pluripotency (Figures S5A–S5D). Overall, these data suggest EMCV-Puro-MYOD1/-mCherry populations have advantages for both uniform transgene activation and myogenic differentiation without showing any advantage of copy number integrations or transfection efficiency.

### Uniform transgene activation in Tet-MyoD hiPSCs depends on sustained *rtTA* expression

**Figure 6:**
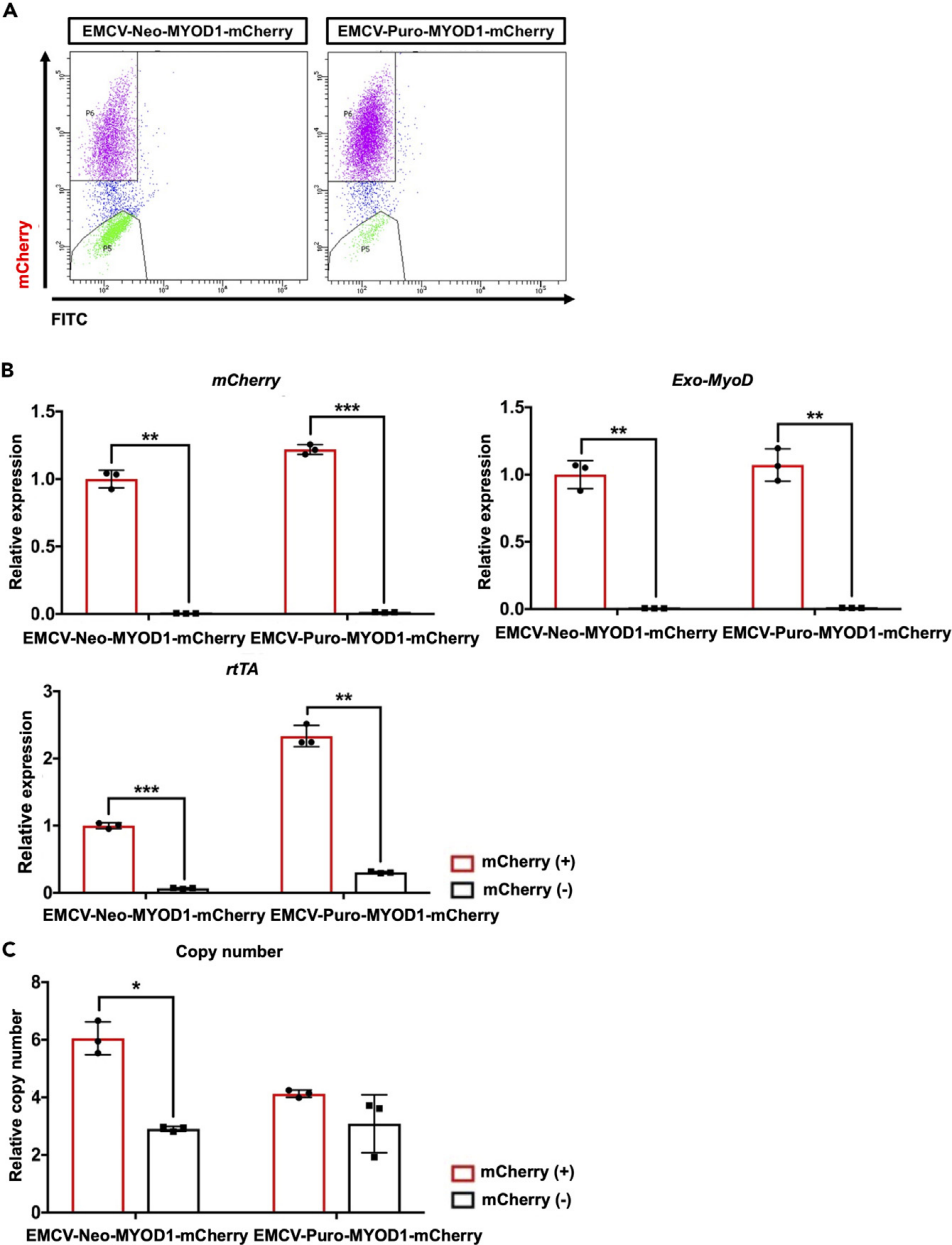
Evaluating transgene expression levels and determining copy number integrations in mCherry-positive and mCherry-negative populations (A) mCherry FACS of Tet-MyoD hiPSCs after 24 h of 1.5 μg/mL dox treatment. Representative graphs are shown. (B) qPCR results showing *mCherry*, *Exo-MyoD*, and *rtTA* expressions in mCherry-positive and mCherry-negative populations sorted from EMCV-Neo-MYOD1-mCherry and EMCV-Puro-MYOD1-mCherry populations. Relative expression levels were normalized to *PBGD* as an internal control in each sample and then to mCherry-positive populations of EMCV-Neo-MYOD1-mCherry. Values represent means ± SD, n = 3. (C) qPCR analysis for Tet-MyoD copy number integrations in mCherry-positive and mCherry-negative populations sorted from Tet-MyoD hiPSCs. Values represent means ± SD, n = 3 ∗p < 0.05, ∗∗p < 0.01, ∗∗∗p < 0.001 according to Paired *t* test.

We also confirmed if lower *rtTA* expression is the main cause of non-transgene activation of mCherry-negative cells in EMCV-Neo-MYOD1-mCherry and EMCV-Puro-MYOD1-mCherry populations after 24 h of dox administration and rescue of its expression can sufficiently improve myogenic differentiation. To this end, first we separated the positive and negative mCherry cells by FACS for subsequent analysis of the transgene expression and Tet-MyoD vector copy number (Figure 6A). The expressions of *mCherry* and *Exo-MyoD* were higher in mCherry-positive populations than those in mCherry-negative populations (Figure 6B) as expected. Expression of *rtTA* was also significantly lower in mCherry-negative populations (Figure 6B) in both EMCV-Neo/Puro-MYOD1-mCherry as we observed in Figure 1.

Although different copy number integrations were observed between mCherry-positive and mCherry-negative populations (Figure 6C), these differences do not represent the expression levels of *rtTA.* In EMCV-Neo-MYOD1-mCherry, while 10 times different *rtTA* expression level was observed between mCherry-positive and negative populations, only 2 times difference was observed for copy number integrations (Figure 6C). In the case of EMCV-Puro-MYOD1-mCherry, while dramatic reduction was observed in *rtTA* expression (Figure 6B), no significant difference was observed in copy number integrations (Figure 6C). Notably, with the extended cultured populations, increased copy number integrations were observed in both mCherry-positive and mCherry-negative cells in EMCV-Neo-MYOD1-mCherry populations (Figure S6A). While almost same copy number integrations were observed between PE 3 of mCherry-positive and PE 8 and 12 of mCherry-negative populations in EMCV-Neo-MYOD1-mCherry (Figure S6A), both *rtTA* and *NeoR* expression levels remained lower in mCherry-negative populations (Figure S6B). Despite the results observed in EMCV-Neo-MYOD1-mCherry populations, both mCherry-positive and mCherry-negative populations in EMCV-Puro-MYOD1-mCherry showed similar copy number integrations among several passages (Figure S6C) and lower *rtTA* and *PuroR* expression levels were observed in the negative populations (Figure S6D). Overall, the data suggest different expression levels of both *rtTA* and antibiotic resistance gene (*NeoR* and *PuroR*) observed between mCherry-positive and mCherry-negative populations are copy number independent.

As we expected previously (Figure 2), simultaneous transfection of EMCV-Neo/Puro-MYOD1-mCherry and an additional CAG-rtTA-EGFP vector (Figure S7A) improved mCherry-positive populations to more than 70% in EMCV-Neo-MYOD1-mCherry populations (Figures S7A and S7B). More than 90% of the GFP-positive population was positive for mCherry in the EMCV-Neo/Puro-MYOD1-mCherry populations (Figures S7C and S7D), suggesting most of the rtTA-supplemented populations improved their Tet-On promoter activity. However, aforementioned percentage is a significant improvement in the number of mCherry-positive cells for EMCV-Neo-MYOD1-mCherry cells; it was not observed in EMCV-Puro-MYOD1-mCherry populations (Figure S7B), which may explained by the fact that EMCV-Puro-MYOD1-mCherry already expressed enough *rtTA* in mCherry-negative populations (Figures 6B and S6D). At the end of the differentiation (d7), improved myogenic differentiation was observed in additional *rtTA*-expressing cells (Figure S8A) as most GFP-positive cells were pan-MHC positive (Figure S8A). Although co-transfection with CAG-rtTA-EGFP significantly improved myogenic differentiation in EMCV-Neo-MYOD1-mCherry cells, this improvement was still less than that in EMCV-Puro-MYOD1-mCherry populations (Figures S7B and S8). Overall, a lower *rtTA* expression appears to be a major cause of Tet-On promoter dysfunction in Tet-MyoD hiPSCs, and rescue of its expression sufficiently improves both transgene expression and myogenic differentiation especially in EMCV-Neo-MYOD1-mCherry populations. As previous report^19^ shows that limited rtTA expression contributes to non-uniform transgene activation in Tet-On systems, our results also suggest that supplementation of rtTA in hiPSCs can contribute to both uniform transgene activation and myogenic differentiation. Finally, sustaining rtTA expression by simply conjugating with puromycin resistance gene would be the most appropriate solution for the uniform transgene activation of Tet-On system.

## Discussion

In this study, we found that the rtTA expression in Tet-On system is critical for activating Tet-On promoter, and thus sustaining its expression by conjugating rtTA with puromycin resistance gene is an effective strategy for achieving uniform transgene activation of both reporter gene mCherry and MYOD1 for myogenic differentiation in polyclonal hiPSCs. Improvement of transgene expression efficiency is one of the major topics in genomic engineering. In agreement with a previous studies of a reprogrammable mouse strain which showed that two copies of the rtTA expression vector could reprogram mouse somatic cells into induced pluripotent stem cells (iPSCs) more efficiently than one copy number^35^ and limited rtTA expression found in non-uniform transgene activation on lentiviral Tet-On systems, which could be improved by rtTA supplementation,^19^ we also found that supplementation with additional *rtTA* could increase myogenic differentiation particularly in G418-selected populations. These results suggest rtTA expression is one of the major causes of non-uniform transgene activation in Tet-On system. However, since we have observed that, after multiple copy number integrations of all-in-one Tet-On system are achieved in hiPSC populations by using *piggyBac* transposon, copy number integrations do not fully represent rtTA expression level in the populations, transgene silencing at safe-harbor locus has been reported,^36^^,^^37^ and rtTA supplementation is often complicated, we sought to investigate new strategy in addition to the current solutions.

It has been revealed that, puromycin-selected cells rather than neomycin-selected cells, achieve higher reporter gene expression in mESCs^20^ and hESCs^21^ after transducing constitutive transgene expressing vector. Indeed, in this study, after transducing all-in-one *piggyBac* Tet-On vector, puromycin-selected populations in hiPSCs showed higher rtTA expression level and achieved uniform transgene activation. Leaky selection of G418 results in populations with lower copy number integrations, or strict selection activity of puromycin enrich populations with higher copy number integrations, which can be one of the possible explanations for different rtTA expression observed between the populations. However, here we would like to conclude that there were no such advantages or bias on the results of using puromycin, based on following results we have obtained. First, puromycin-selected populations showed lower copy number integrations of *piggyBac* Tet-mCherry/MYOD1 vector, comparing to those of G418-selected populations. Second, multiple copy number integrations have been observed in mCherry-positive and mCherry-negative cells of both neomycin- and puromycin-selected populations. Third, increased copy number integrations have been observed during several passages of neomycin-selected populations, indicating non-integrated populations no longer remained in the populations and thus there should be no leaky selection of G418. Comparing to puromycin selection, G418 selection usually eliminates cell slowly. However, continuous G418 selection, at least around 84 days of selection in this study, has never improved transgene activation or myogenic differentiation in the populations, suggesting selection speed is also not a main explanation of the results. Explanation for two different antibiotic-selected populations showing different rtTA expression levels needs more discussion other than their antibiotic selection activities. Still, it is possible that in the puromycin-selected populations, majority of *piggyBac* vectors are likely to be adopted to the chromosomal position, which is more favorable to express integrated transgene. Future study of single genome targeting insertion may be required as it may avoid possible bias of chromosomal positions in polyclonal Tet-On hiPSCs.

Usually, in bicistronic expression vector with IRES, first transgene shows higher protein expression level than second transgene, which may be explained by different translation efficiency between CAP-dependent or IRES-dependent translation.^33^ For transcription, the first and second transgene are transcribed as one fusion mRNA with IRES, and thus it can be predicted that they are expressed with the same efficiency. In this study, as rtTA is conjugated to antibiotic resistance genes with IRES, we observed similar expression levels between *rtTA* and *NeoR* or *PuroR*. However, both *rtTA* and antibiotic resistance gene of *NeoR* expression levels are much lower in G418-selected populations than those in puromycin-selected populations. After sorting out mChery-positive populations from G418- and puromycin-selected populations, it has been revealed that *rtTA* and *PuroR* were highly transcribed. This suggests that fusion mRNA of *rtTA* and *PuroR* is more favorable to be expressed than *NeoR.* Recently, it has been reported that adjacent transcriptional regions (ATRs) influence protein expression of polycistronic expression vector.^38^ It would be a great interest to study whether unique sequences in the NeoR and PuroR themselves could distinctively influence transcription process including mRNA stability, efficiency of mRNA synthesis, or epigenetic modifications on the promoter, as previously reported.^39^ Although most of the puromycin-selected populations achieved higher myogenic differentiation, there are few populations that remain to be improved. To achieve such improvement, further examination of MYOD1 expression or preventing Tet-On promoter activity from gene silencing may be required as previously reported.^40^^,^^41^^,^^42^ Despite using similar construct, rtTA linked with puromycin resistance gene, and non-uniform transgene activation in lentiviral Tet-On system has been reported.^19^ As the study suggests, this could be explained by potential gene-silencing events with lentiviral vector,^19^ and *piggyBac* transposon vector with puromycin resistance gene would be one of the most promising construct for Tet-On system.

Overall, in this study we demonstrate efficacy of upregulating rtTA expression for uniform transgene activation in Tet-On system. We also achieved uniform transgene activation by simply swapping the neomycin resistance gene to a puromycin resistance gene in our previous all-in-one Tet-On system, which also led to a higher efficiency of myogenic differentiation of hiPSCs without any need for prior clonal evaluation or rtTA supplementation. Remarkably, significant improvement of transgene activation by the rtTA supplementation was only observed in G418-selected populations, suggesting rtTA expression can be sufficiently sustained by simply connecting to puromycin resistance gene. Our findings would save researchers from resource and time spending on obtaining stable transgene expressing clones for studying specific transgene or directed differentiation of skeletal muscle cells as performed here but may have broader implications to Tet-On-based systems applied to reprogramming or cell lineages derivation achieved by the inducible overexpression of transcription factors.

### Limitations of the study

Although our study has demonstrated that swapping neomycin resistance gene with puromycin resistance gene achieves uniform transgene activation by upregulating IRES-linked rtTA expression, the random integration of *piggyBac* vector may be considered as potential biases on the transgene expression. Thus, single genomic targeting insertion of the vector constructs would be required in future studies to confirm that puromycin resistance gene upregulates IRES-linked rtTA expression without any possible biases from genomic loci affecting transgene expression. Finally, the unique gene sequence of the selection markers might have an influence on the IRES-linked transgene expression.

## STAR★Methods

### Key resources table

**Table:** 

REAGENT or RESOURCE	SOURCE	IDENTIFIER
**Experimental models: Cell lines**		

HC #1 hiPS cell lines	Sasaki-Honda et al.^17^	Graduate School and Faculty of Medicine of Kyoto University (approval numbers #R0091)

**Oligonucleotides**		

*Msc*I-*Eag*I adapter oligos (Fwd: CCACAACCATGA TTGAACAAGATGGATTGCACGCAGGTTCTCC)	This paper	N/A
*Msc*I-*Eag*I adapter oligos (Rev: GGCCGGAGAACCTG CGTGCAATCCATCTTGTTCAATCATGGTTGTGG)	This paper	N/A

**Recombinant DNA**		

PB-TA-ERN	Laboratory of Knut Woltjen (Kim et al.)^11^	Addgene Plasmid #80474, KW110
PB-TAC-ERN	Laboratory of Knut Woltjen (Kim et al.)^11^	Addgene Plasmid #80475, KW111
PB-TA-ERP2	Laboratory of Knut Woltjen (Kim et al.)^11^	Addgene Plasmid #80477, KW542
PB-TAC-ERP2	Laboratory of Knut Woltjen (Kim et al.)^11^	Addgene Plasmid #80478, KW543
PB-TA-ERN2	This paper	KW1406
PB-TAC-ERN2	This paper	KW1413
PB-CAG-rtTA-ires-Puro	Collinson et al.^43^	N/A
pENTR-MyoD	Tanaka et al.^5^	N/A
EMCV-Neo-MYOD1 (PB-TA-ERN2-MyoD)	This paper	KW1410
EMCV-Neo-MYOD1-mCherry (PB-TAC-ERN2-MyoD)	This paper	KW1415
EMCV-Puro-MYOD1-mCherry (PB-TAC-ERP2)	This paper	KW1409
FMDV-Neo-MYOD1-mCherry (PB-TAC-ERN-MyoD)	Tanaka et al.^5^	KW698
EMCV-Puro-MYOD1 (PB-TA-ERP2-MyoD)	Uchimura et al.^12^	KW879
CAG-rtTA-EGFP (PB-CAG-rtTA-ires-GFP)	This paper	KW1480
p5E-CAG	Laboratory of Knut Woltjen	KW140
pENTR-rtTA-Adv	Laboratory of Knut Woltjen	KW555
p3E-IRES-EGFPpA	Kwan et al.^44^	CC389
pDestPB53	Laboratory of Knut Woltjen	KW136
EMCV-Puro-mCherry (PB-TA-ERP2-MyoD)	This paper	KW1561
EMCV-Neo-mCherry (PB-TA-ERN2-mCherry)	This paper	KW1562
CAG-EGFP (PB-GFPa)	Laboratory of Knut Woltjen	KWan091
*piggyBac* transposase expression vector (pCAG-PBase)	Laboratory of Knut Woltjen, (Kim et al.)^11^	KW158

**Software and algorithms**		

BD FACS Diva software	BD Biosciences	N/A
FlowJo software	Tree Star	N/A
Endmemo	http://www.endmemo.com/index.php	N/A
GraphPad Prism	GraphPad	N/A
BZ-X710	Keyence	N/A

### Resource availability

#### Lead contact

Further information and requests for resources and reagents should be directed to and will be fulfilled by the Lead Contact, Hidetoshi Sakurai (hsakurai@cira.kyoto-u.ac.jp).

#### Materials availability

All unique/stable reagents generated in this study are available from lead contact, Hidetoshi Sakurai, with a completed Material Transfer Agreement.

### Experimental model and study participant etails

#### Human iPSC lines

HC #1 hiPS cell lines served as healthy controls. HC #1 described in key resources table. HC #1 hiPSCs line was used in following approval by the Ethics Committee of the Graduate School and Faculty of Medicine of Kyoto University (approval numbers #R0091). Karyotype was evaluated by G-banding method in LSI Medience.

#### Feeder-free hiPSC culture

Tet-mCherry or Tet-MyoD hiPSCs were cultured on Easy iMatrix-511 silk-coated plates (#892024, Nippi) in StemFit medium (AK02N, Ajinomoto) containing 100 μg/mL G418 (#938044, NacalaiTesque) or 0.5 μg/mL puromycin dihydrochloride (160-23151, Wako Chemicals). Cells were passaged every 7 days using Accutase (#12679-54, NacalaiTesque) and seeded on Easy iMatrix-511 silk-coated 6-well plates in the presence of 10 μM Y-27632 (NacalaiTesque) at a density of 1.5×104 cells/well for the first 2 days after plating. At 48 h after passaging, Y-27632 was removed and replaced with StemFit medium containing the appropriate antibiotic.

### Method details

#### Plasmid construction

FMDV-Neo *piggyBac* Gateway Destination vectors PB-TA-ERN (KW110) and PB-TAC-ERN (KW111), and EMCV-Puro *piggyBac* Gateway Destination vectors PB-TA-ERP2 (KW542) and PB-TAC-ERP2 (KW543) were described in key resources table. EMCV-Neo *piggyBac* Gateway Destination vectors PB-TA-ERN2 (KW1406) and PB-TAC-ERN2 (KW1413) were constructed by replacement of the FMVD IRES sequence with an *Fse*I-*Msc*I EMCV IRES fragment from PB-CAG-rtTA-ires-Puro (a gift from Dr. Jonathan Draper) 42 and annealed *Msc*I-*Eag*I adapter oligos (Fwd: CCACAACCATGATTGAACAAGATGGATTGCACGCAGGTTCTCC, Rev: GGCCGGAGAACCTGCGTGCAATCCATCTTGTTCAATCATGGTTGTGG). MyoD expression vectors were constructed by Gateway cloning of the *MYOD1* cDNA from pENTR-MyoD 5 into the series of *piggyBac* Gateway Destination vectors described above, resulting in EMCV-Neo-MYOD1 (PB-TA-ERN2-MyoD, KW1410), EMCV-Neo-MYOD1-mCherry (PB-TAC-ERN2-MyoD, KW1415), and EMCV-Puro-MYOD1-mCherry (PB-TAC-ERP2, KW1409). The FMDV-Neo-mCherry (PB-TAC-ERN-MyoD, KW698) and EMCV-Puro (PB-TA-ERP2-MyoD, KW879) MyoD expression vectors were constructed by Gateway cloning of the *MYOD1* cDNA into KW111 and KW542.

PB-CAG-rtTA-ires-GFP (KW1480) was constructed using Multisite Gateway cloning of p5E-CAG (KW140; Kim et al. in review), pENTR-rtTA-Adv (KW555) and p3E-IRES-EGFPpA (CC389; Kwan et al., 2007^44^) into pDestPB53 (KW136; Kim et al. in review).

#### Generating Tet-mCherry and Tet-MyoD hiPSCs with customized Tetracycline inducible vectors

To generate Tet-mCherry or Tet-MyoD hiPSCs, 5.0 μg of Tet-mCherry (EMCV-Neo/Puro-mCherry) or Tet-MyoD vectors and 5.0 μg of *piggyBac* transposase expression vector were electroporated into 1×106 hiPSCs by using a NEPA21 electroporator (Nepagene). Briefly, hiPSCs were treated with 10 μM Y-27632 1 day before electroporation. Cells were dissociated into single cells with accutase, and 1.0x106 cells were resuspended in Opti-MEM. Tet-mCherry or Tet-MyoD vectors with *pigguBac* transposase expression vector were electroporated under the conditions described in Table S1. The electroporated cells were plated on Easy iMatrix-511 silk-coated 6-well plates at 2.0×105 cells per well in StemFit medium containing Y-27632. Selection with 100 μg/mL G418 or 0.5 μg/mL puromycin was started 48 h after the electroporation. After 5 days of antibiotic selection, the cells were passaged with continuous antibiotic selection.

#### Direct skeletal muscle cell differentiation

Skeletal muscle cell differentiation of Tet-MyoD hiPSCs was performed as following. Briefly, 3.0×105 cells were seeded on Matrigel-coated (#356231, BD Biosciences) 6-well plates (1:100) in StemFit medium supplemented with 10 μM Y-27632. At 24 h after seeding, the medium was changed to primate embryonic stem cell medium (RCHEMD001, ReproCELL) without Y-27632. After 24 h, 1.5 μg/mL dox (LKT Laboratories) was added to the culture medium. After an additional 24 h, the medium was changed into differentiation medium composed of α-MEM (with L-Gln, ribonucleosides, and deoxyribonucleosides, #21444-05, NacalaiTesque) with 5% KSR (#10828028, Invitrogen), 5% Penicillin Streptomycin Mixed solution (#09367-34, NacalaiTesque), 200 μM 2-mercaptoethanol and 1.5 μg/mL dox. Culturing was continued until day 7 with daily medium changes for immunofluorescence microscopy.

#### Quantification of skeletal muscle cell differentiation

The efficiency of skeletal muscle cell differentiation was analyzed using Keyence software. The total number of nuclei and MHC-positive nuclei were counted. The differentiation efficiency was then calculated by dividing the number of MHC-positive nuclei by the total number of nuclei.

#### Immunofluorescence microscopy

To identify differentiated cells, myogenic markers were used for immunostaining. Differentiated cells were fixed with 4% or 2% PFA/DPBS (-) at 4°C for 10 min. After being washed in DPBS (-), the cells were treated with methanol:H2O2 (100:1) at 4°C for 15 min (in the case of d7 differentiated cells only) and subsequently blocked with Blocking One (#03953-95, NacalaiTesque) at 4°C for 45 min. The cells were then incubated with primary antibody in 10% Blocking One in DPBS (-) with 0.2% Triton X-100 (Santa Cruz Biotechnology) at 4°C overnight. Next, the samples were washed three times with DPBS (-) containing 0.2% Triton X-100. The cells were then incubated at room temperature for 1 h with secondary antibody in 10% Blocking One in DPBS (-) containing 0.2% Triton X-100. DAPI (1:5000) was used to counter-stain the nuclei. The samples were observed under a BZ-X710 fluorescence microscope (Keyence).

For pluripotent marker immunostaining, the hiPSCs were fixed with 4% PFA/DPBS (-) at 4°C for 10 min. After being washed in DPBS (-), the cells were blocked with Blocking One at 4°C for 45 min. The cells were then incubated at 4°C overnight with primary antibody in 10% Blocking One in DPBS (-) containing 0.2% Triton X-100. Next, the samples were washed three times with DPBS (-) containing 0.2% Triton X-100. The cells were then incubated at room temperature for 1 h with secondary antibody in 10% Blocking One in DPBS (-) and 0.2% Triton X-100. DAPI (1:5000) was used to counter-stain the nuclei. The samples were observed under a BZ-X710 microscope at 200× magnification. The primary and secondary antibodies used in this study are listed in Table S2.

#### FACS

To isolate mCherry-positive and mCherry-negative populations of Tet-mCherry or Tet-MyoD hiPSCs, the cells were harvested as a single cell suspension in HBSS buffer using Accutase and filtered through a cell strainer. Cells were analyzed and sorted on a FACS Aria II (BD Biosciences).

#### Co-transfection of Tet-MyoD vector with additional rtTA or EGFP expression vector

For co-transfection, 5.0 μg Tet-MyoD vectors and *piggyBac* transposase expression vector with CAG-rtTA-EGFP or CAG-EGFP were electroporated simultaneously into hiPSCs using a NEPA21 electroporator. In total, 15 μg of plasmid vectors were electroporated into hiPSCs.

#### Flow cytometry

To measure mCherry or GFP reporter fluorescence, Tet-mCherry or Tet-MyoD hiPSCs were suspended in HBSS buffer and analyzed using a BD LSR FortessaTM Cell Analyzer (BD Biosciences) with BD FACS Diva software (BD Biosciences). Hoechst staining (1:2000) was used to exclude dead cells. Data were analyzed and generated by FlowJo software (Tree Star).

#### RNA isolation and reverse transcription

Total RNA was isolated using the ReliaPrep RNA cell Miniprep Kit System (Z6012, Promega) according to the manufacturer’s instructions. Residual genomic DNA was digested and removed using DNase I (Promega) treatment. First-strand cDNA was generated from extracted total RNA using ReverTra Ace qPCR RT Master Mix with gDNA remover (FSQ301, TOYOBO). The qPCR was performed using Power SYBR Green (#4368708, Applied Biosystems) and the Step One Plus thermal cycler (Applied Biosystems). All samples were normalized to the house-keeping gene Porphobilinogen Deaminase 1 (*PBGD*) 6. For absolute quantification of transgene expression levels, the plasmid backbone of the EMCV-Neo/Puro-MYOD1-mCherry vectors were used to construct a standard curve with serial dilutions of 1/10 for qPCR analysis and estimation of the plasmid copy number was calculated using the web server Endmemo (http://www.endmemo.com/index.php). Each transgene’s copy number was estimated by annotating its cycle threshold (Ct) value and comparing it to the standard curve of the plasmid copy number. The primer sets used in this study are listed in Table S3.

#### Genomic DNA isolation and copy number integration analysis

Genomic DNA was extracted from mCherry-positive and mCherry-negative populations of Tet-mCherry or Tet-MyoD hiPSCs after sorting. Genomic DNA was extracted using the GenElute Mammalian Genomic DNA Miniprep Kit (Sigma-Aldrich) according to the manufacturer’s instructions. Copy number analysis was performed by qPCR as specified above, using Power SYBR Green and the Step One Plus thermal cycler. Approximately, 8 ng of extracted genomic DNA was used for each qPCR. rtTA primer was used for detecting the Tet-mCherry or Tet-MyoD vector and DLX5 was used as internal control. A single knocked-in Tet-MyoD vector cell line served as a single copy number control.

### Quantification and statistical analysis

For all experiments, data are reported as the mean ± SD. For comparison of two samples, p value were analyzed using either an unpaired Student’s *t*-test or Paired *t*-test. For comparison of multiple samples, p value were analyzed using one-way ANOVA followed by Tukey’s test or Dunnett’s test. The above p value were analyzed using GraphPad Prism.

## Data Availability

• Data: The data generated and analyzed during the current study are available from the lead contact upon reasonable request. • Code: This study did not generate/analyze [dataset/code]. • Other items: Any additional information to reanalyze the data reported in this study is available from the lead contact upon reasonable request.
