# Supplementary material for: Uniform transgene activation in Tet-On systems depends on sustained rtTA expression
Source: iScience. 2023 Aug 19;26(10):107685. doi: 10.1016/j.isci.2023.107685 (PMC10494183; doi:10.1016/j.isci.2023.107685)
Supplement: Document S1. Figures S1–S8 and Tables S1–S3 [file mmc1.pdf]

iScience, Volume *26*

Supplemental information

Uniform transgene activation in Tet-On systems depends on sustained rtTA expression

Jun Otomo, Knut Woltjen, and Hidetoshi Sakurai

Fig S1

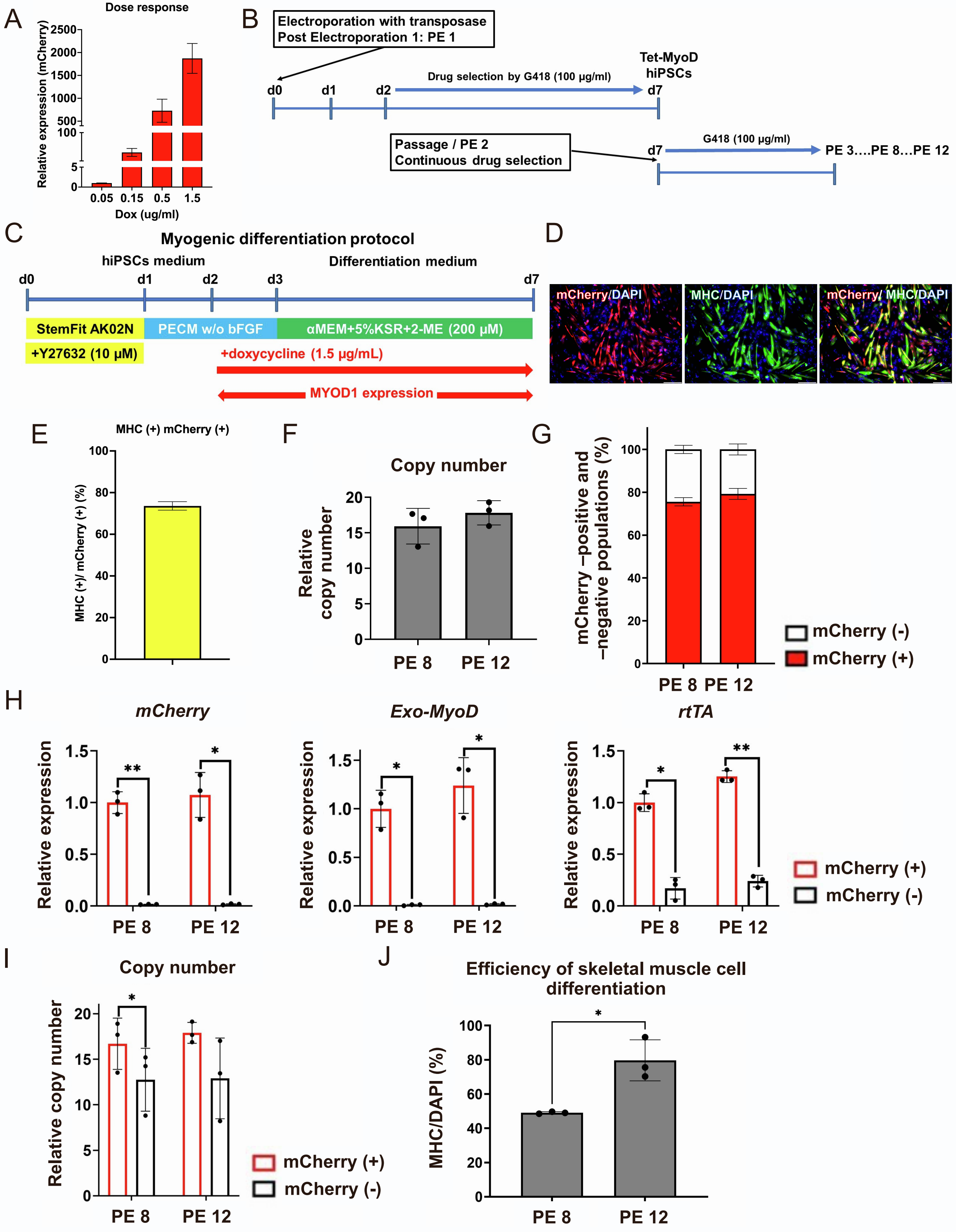

A
Dose response
Relative expression (mCherry)
Dox (ug/ml)
0.05 0.15 0.5 1.5
B
Electroporation with transposase
Post Electroporation 1: PE 1
Drug selection by G418 (100 μg/ml)
Tet-MyoD hiPSCs
d0 d1 d2 d7
Passage / PE 2
Continuous drug selection
G418 (100 μg/ml)
PE 3….PE 8…PE 12
C
Myogenic differentiation protocol
hiPSCs medium
Differentiation medium
d0 d1 d2 d3 d7
StemFit AK02N
PECM w/o bFGF
αMEM+5%KSR+2-ME (200 μM)
+Y27632 (10 μM)
+doxycycline (1.5 μg/mL)
MYOD1 expression
D
mCherry/DAPI
MHC/DAPI
mCherry/ MHC/DAPI
E
MHC (+) mCherry (+)
MHC (+)/ mCherry (+) (%)
F
Copy number
Relative copy number
PE 8 PE 12
G
mCherry –positive and –negative populations (%)
mCherry (-)
mCherry (+)
PE 8 PE 12
H
mCherry
Exo-MyoD
rtTA
Relative expression
PE 8 PE 12
mCherry (+)
mCherry (-)
I
Copy number
Relative copy number
PE 8 PE 12
mCherry (+)
mCherry (-)
J
Efficiency of skeletal muscle cell differentiation
MHC/DAPI (%)
PE 8 PE 12

Figure S1. Generation, characterization and evaluation of Tet-MyoD hiPSCs with prolonged culture, related to Fig 1.

(A) qPCR result showing dose response expression of mCherry. Expression of mCherry has been evaluated among 4 different doses of dox treatment (1.5, 0.5, 0.15, 0.05 μg/ml) on FMDV-Neo-MYOD1-mCherry hiPSCs. The samples were collected after 24h of dox treatment. Relative expression levels were normalized to *PBGD* as an internal control in each sample. Values represent means ± SD, n=3 (technical replicates). **(B)** Scheme for generating Tet-MyoD hiPSCs and subsequent analysis. Electroporated cells were seeded on laminin 511-coated plates. Antibiotic (G418) selection began 48 h after electroporation and cells were cultured under G418 pressure. PE (Post Electroporation) indicates number of passages after the electroporation. Cells were cultured up to PE 12 and subsequent analysis were conducted at each passage of PE 3 (Fig 1), PE 8 and PE12. **(C)** Schematic representation of the directed myogenic differentiation from Tet-MyoD hiPSCs. Cells were reseeded on Matrigel-coated plates, and after 48 h, 1.5 μg/mL dox was added. For the first 3 days, the cells were cultured in hiPSCs medium (StemFit AK02N or primate embryonic stem cell medium (PECM)) and then in differentiation medium. Myogenic differentiation was completed by day 7 (d7). **(D)** Immunostaining for mCherry fluorescence (Red) and the skeletal muscle cell marker MHC (Green) images. DAPI (blue) was used for counter-staining. Differentiated cells were fixed and stained on day7 of the differentiation. Scale bars, 200 μm **(E)** Quantification of double positive populations for MHC and mCherry. Ratio was calculated by counting the total number of mCherry (Red)/MHC (Green) stained cells and then dividing by the number by total number of mCherry cells. Values represent means ± SD, n=3 (technical replicates).**(F)** qPCR analysis for Tet-MyoD copy number integrations in generated FMDV-Neo-MYOD1-mCherry populations at PE 8 and PE 12. Values represent means ± SD, n=3. **(G)** Quantification of mCherry-positive (red) and mCherry-negative populations (white) from the FCM analysis of FMDV-Neo-MYOD1-mCherry populations at PE 8 and PE 12. Values represent means ± SD, n=3. **(H)** qPCR results showing *Exo-MyoD*, *mCherry*, and *rtTA* expressions in mCherry-positive and mCherry-negative populations sorted from FMDV-Neo-MYOD1-mCherry populations at PE 8 and PE 12. **(I)** qPCR analysis for Tet-MyoD copy number integrations in mCherry-positive and mCherry-negative populations sorted from FMDV-Neo-MYOD1-mCherry populations at PE 8 and PE 12. **(J)** Quantification of skeletal muscle cell differentiation efficiency at PE 8 and PE 12 of FMDV-Neo-MYOD1-mCherry populations. Values represent means ± SD, n=3. *P<0.05 **P<0.01 according to Student's *t*-test (unpaired) in (F), (G) and (J), Paired *t*-test in (H) and (I).

Fig S2

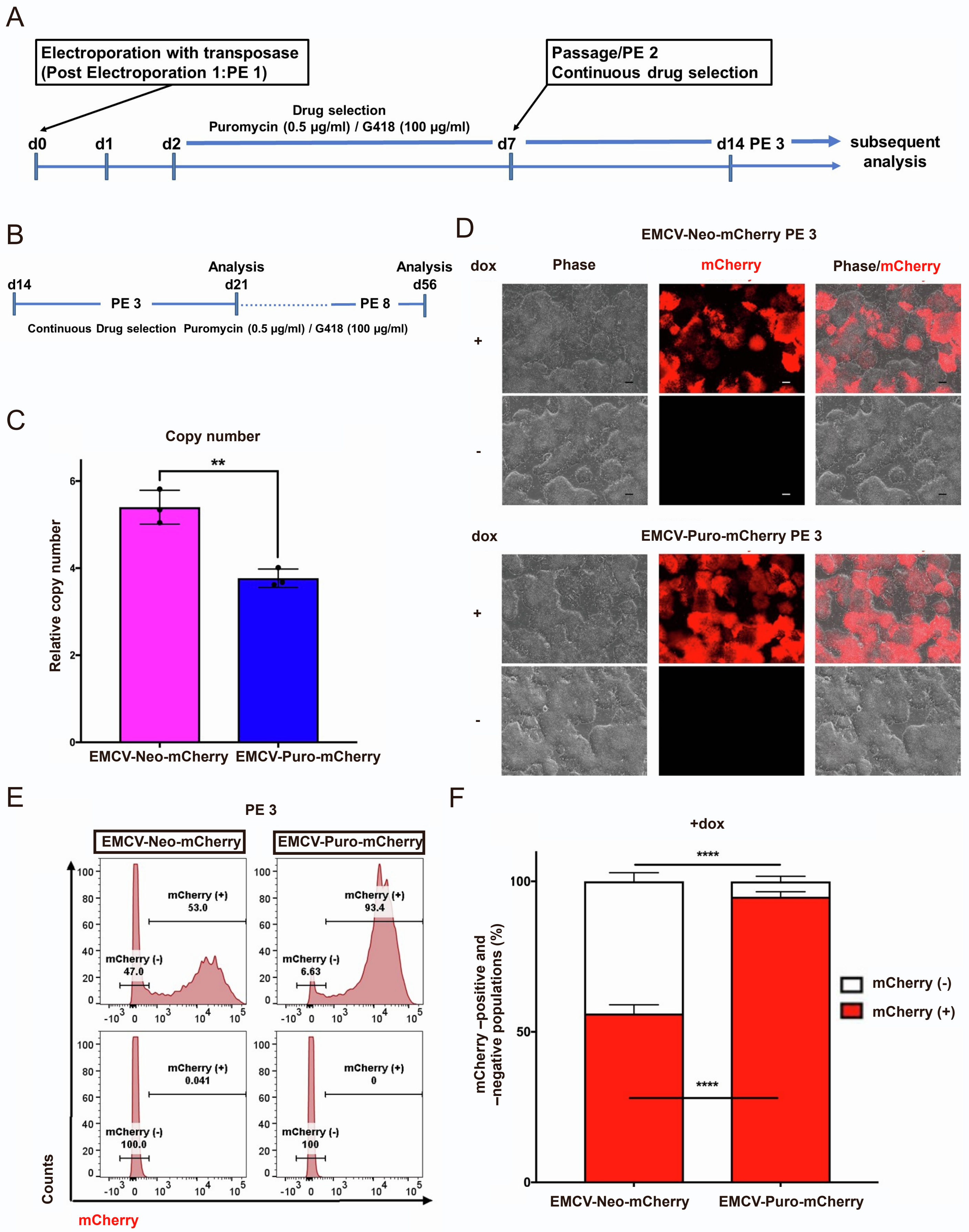
A
Electroporation with transposase
(Post Electroporation 1:PE 1)
Passage/PE 2
Continuous drug selection
Drug selection
Puromycin (0.5 μg/ml) / G418 (100 μg/ml)
d0
d1
d2
d7
d14 PE 3
subsequent analysis
B
Analysis
Analysis
d14
d21
d56
PE 3
PE 8
Continuous Drug selection Puromycin (0.5 μg/ml) / G418 (100 μg/ml)
C
Copy number
**
Relative copy number
EMCV-Neo-mCherry
EMCV-Puro-mCherry
D
EMCV-Neo-mCherry PE 3
dox
Phase
mCherry
Phase/mCherry
+
-
dox
EMCV-Puro-mCherry PE 3
+
-
E
PE 3
EMCV-Neo-mCherry
EMCV-Puro-mCherry
mCherry (+)
53.0
mCherry (-)
47.0
mCherry (+)
93.4
mCherry (-)
6.63
mCherry (+)
0.041
mCherry (-)
100.0
mCherry (+)
0
mCherry (-)
100
Counts
mCherry
F
+dox
****
****
mCherry –positive and –negative populations (%)
mCherry (-)
mCherry (+)
EMCV-Neo-mCherry
EMCV-Puro-mCherry

Figure S2. Generation of Tet-mCherry hiPSCs and evaluation of their transgene expression, related to Fig 3.

(A) Scheme for generating Tet-mCherry hiPSCs and subsequent analysis. Electroporated cells were seeded on laminin 511-coated plates. Antibiotic selection (G418 or Puromycin) began 48 h after electroporation and cells were cultured under antibiotic pressure. PE (Post Electroporation) indicates number of passages after the electroporation. **(B)** Cells were cultured up to PE 8 and subsequent analysis were conducted at each passage of PE 3 and PE 8 (Fig 3, 4). **(C)** qPCR analysis for Tet-mCherry copy number integrations in generated Tet-mCherry hiPSCs at PE 3. The rtTA primer was used for detecting Tet-mCherry and Tet-MyoD vectors and DLX5 was used as an internal control. A single copy number integrated Tet-MyoD vector cell line was used as the reference. Values represent means ± SD, n=3. **(D)** Phase-contrast and fluorescence images showing mCherry expression in the generated Tet-mCherry hiPSCs. Images were taken after 24 h of dox treatment (top row) or without dox (bottom row). Scale bars, 200 µm. **(E)** mCherry FCM analysis of EMCV-Neo-mCherry and EMCV-Puro-mCherry populations at PE 3, after 24 h of 1.5 µg/mL dox treatment (top row) or without dox (bottom row). Representative graphs are shown. **(F)** Quantification of mCherry-positive (red) and mCherry-negative populations (white) from the FCM data in Figure S2E. **P<0.01, ****P<0.0001 according to Student's *t*-test (unpaired).

Fig S3

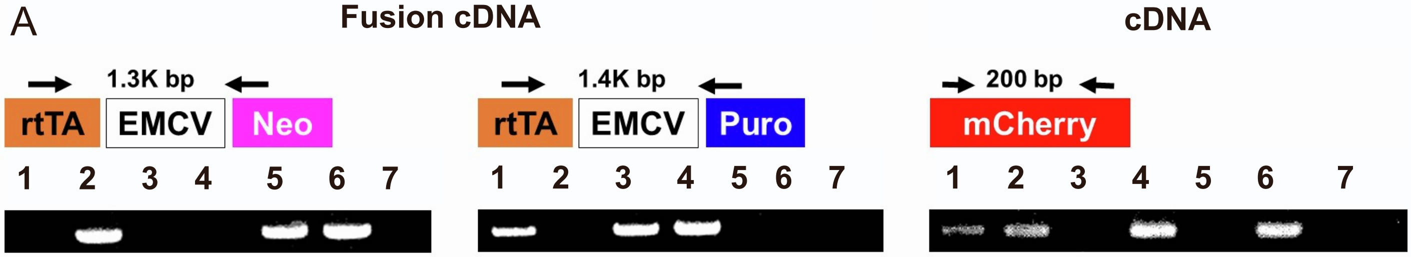

1: EMCV-Puro-mCherry vector
2: EMCV-Neo-mCherry vector
3: EMCV-Puro-mCherry –dox
4: EMCV-Puro-mCherry +dox
5: EMCV-Neo-mCherry –dox
6: EMCV-Neo-mCherry +dox
7: H2O

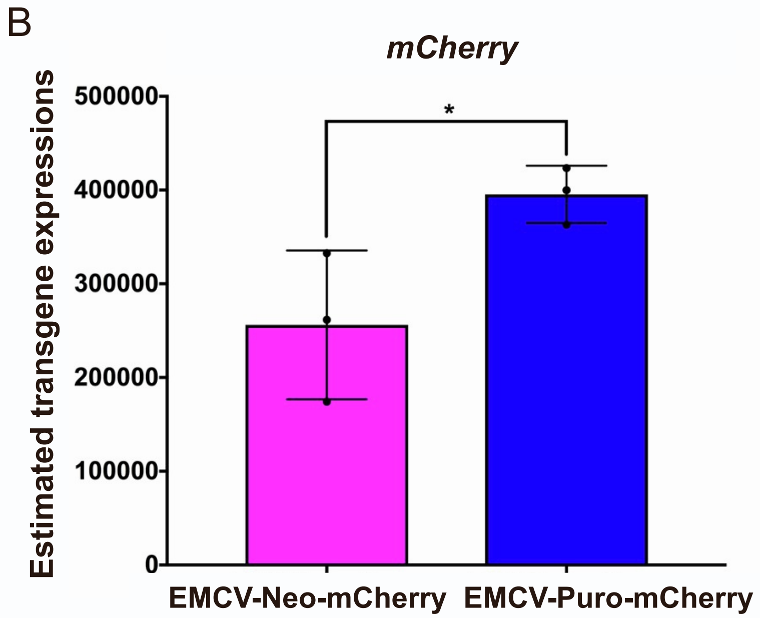

Figure S3. RT-PCR for detecting fusion transcription in Tet-mCherry hiPSCs, related to Fig 3 and 4.

(A) RT-PCR was performed for *rtTA-NeoR*, *rtTA-PuroR* and *mCherry* expressions. **(B)** qPCR results showing *mCherry* expressions in mCherry-positive populations sorted from EMCV-Neo-mCherry and EMCV-Puro-mCherry populations. A standard curve was constructed for each transgene by serially diluting the plasmid vector. Data were normalized to the amount of total RNA (15 ng) used for the cDNA synthesis. Values represent means ±SD, n=3. *P<0.05 according to Student's *t*-test (unpaired).

Fig S4

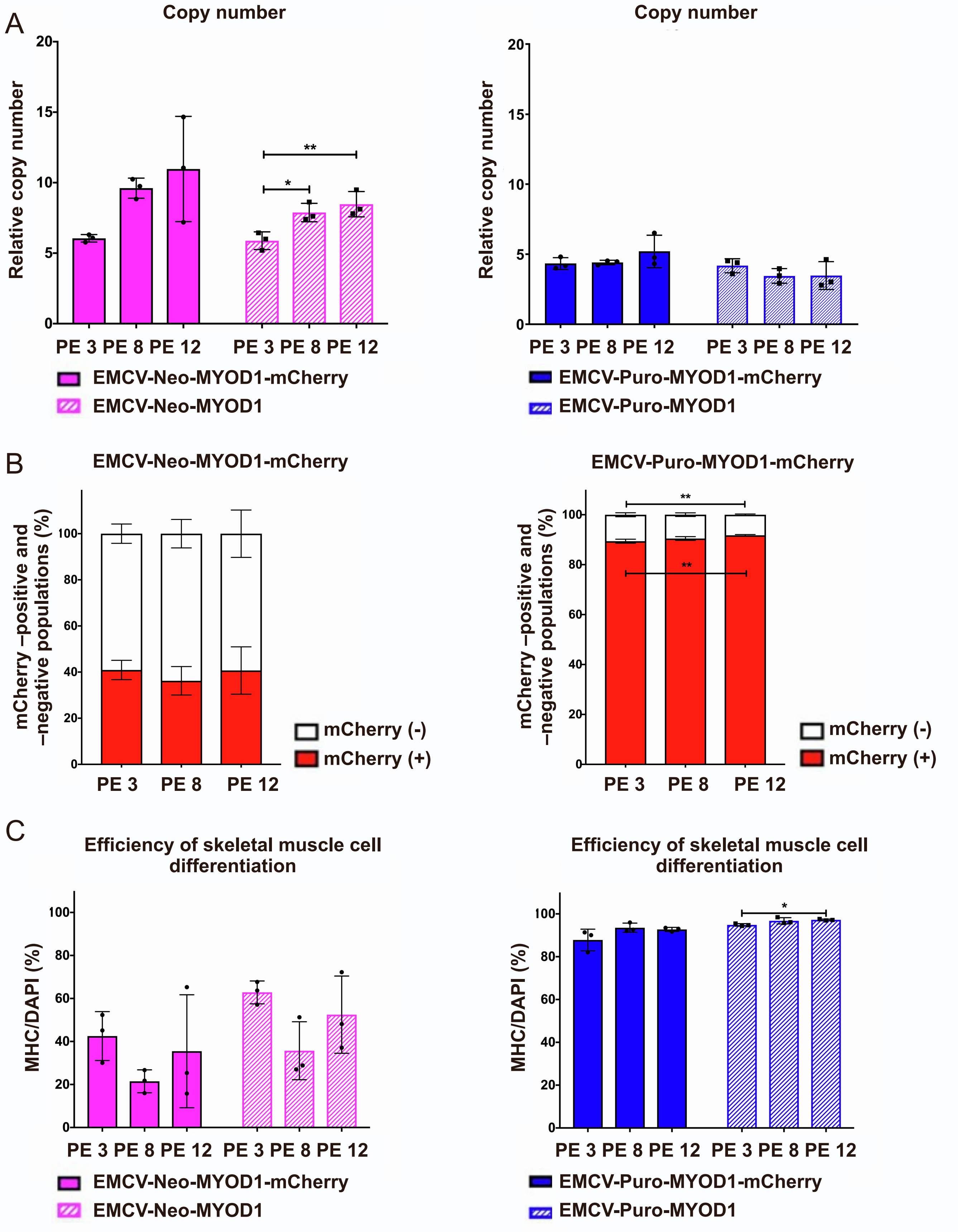
A
Copy number
Relative copy number
20
15
10
5
0
**
*
PE 3 PE 8 PE 12 PE 3 PE 8 PE 12
EMCV-Neo-MYOD1-mCherry
EMCV-Neo-MYOD1
Copy number
Relative copy number
EMCV-Puro-MYOD1-mCherry
EMCV-Puro-MYOD1
B
EMCV-Neo-MYOD1-mCherry
mCherry –positive and –negative populations (%)
100
80
60
40
20
0
PE 3
PE 8
PE 12
mCherry (-)
mCherry (+)
EMCV-Puro-MYOD1-mCherry
**
**
C
Efficiency of skeletal muscle cell differentiation
MHC/DAPI (%)
EMCV-Neo-MYOD1-mCherry
EMCV-Neo-MYOD1
EMCV-Puro-MYOD1-mCherry
EMCV-Puro-MYOD1
*

Figure S4. Evaluation of Tet-MyoD hiPSCs with prolonged culture, related to Fig 5.
(A) qPCR analysis for Tet-MyoD copy number integrations in generated Tet-MyoD hiPSCs at PE 3, PE8 and PE 12. The rtTA primer was used for detecting Tet-MyoD vectors and DLX5 was used as an internal control. A single copy number integrated Tet-MyoD vector cell line was used as the reference. Values represent means ± SD, n=3. **(B)** Quantification of mCherry-positive (red) and mCherry-negative populations (white) of EMCV-Neo-MYOD1-mCherry and EMCV-Puro-MYOD1-mCherry at PE 3, PE 8 and PE 12 based on FCM analysis. Values represent means ± SD, n=3. **(C)** Quantification of skeletal muscle cell differentiation efficiency of the Tet-MyoD hiPSCs at PE 3, PE 8 and PE 12. Efficiency was calculated by counting the total number of nuclei (blue) in MHC-positive cells (green) and then dividing the number by the total number of nuclei. Values represent means ± SD, n=3. *P<0.05 **P<0.01 according to one-way ANOVA followed by Dunnett's test.

Fig S5

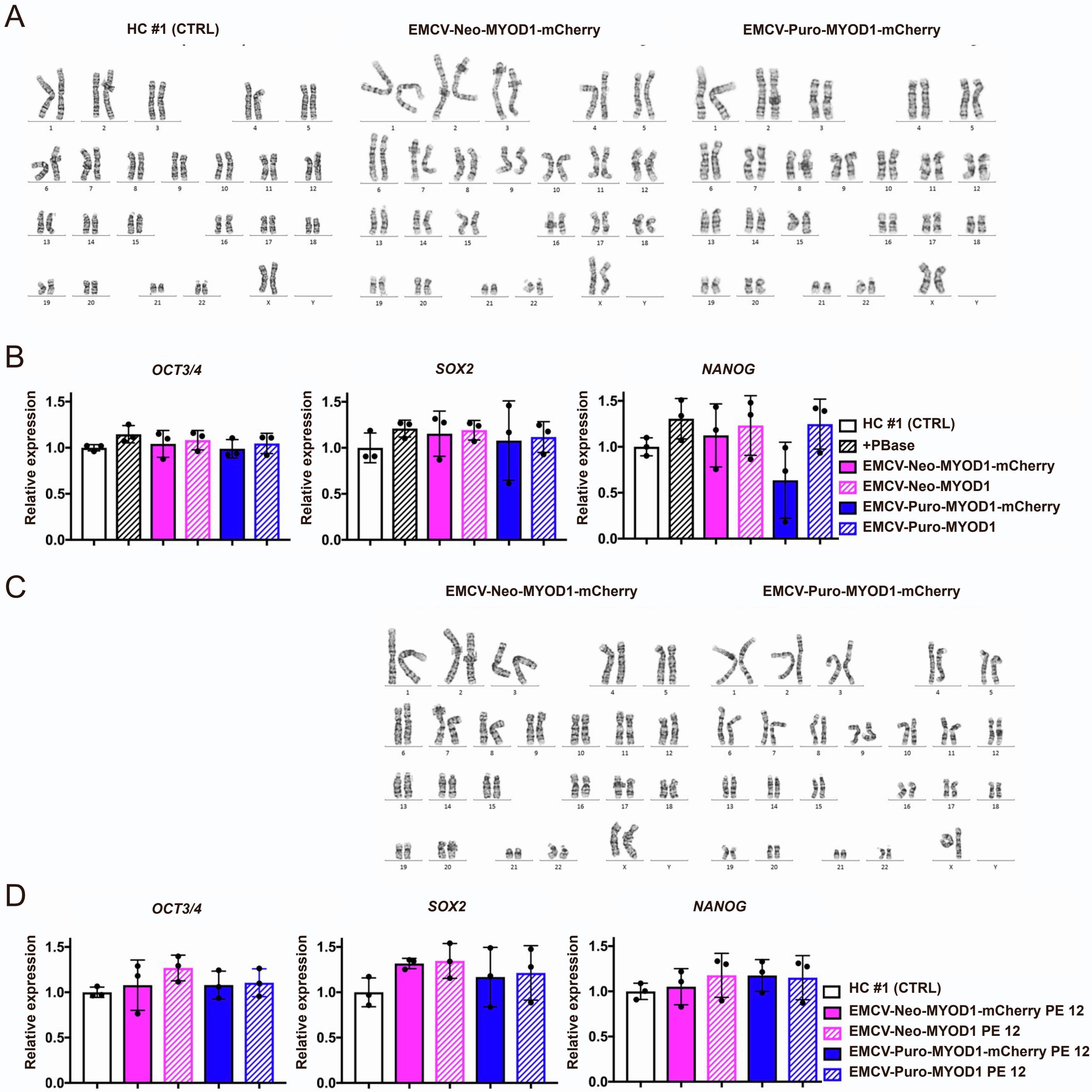
A
HC #1 (CTRL)
EMCV-Neo-MYOD1-mCherry
EMCV-Puro-MYOD1-mCherry
B
OCT3/4
SOX2
NANOG
Relative expression
0.0
0.5
1.0
1.5
HC #1 (CTRL)
+PBase
EMCV-Neo-MYOD1-mCherry
EMCV-Neo-MYOD1
EMCV-Puro-MYOD1-mCherry
EMCV-Puro-MYOD1
C
EMCV-Neo-MYOD1-mCherry
EMCV-Puro-MYOD1-mCherry
D
OCT3/4
SOX2
NANOG
Relative expression
HC #1 (CTRL)
EMCV-Neo-MYOD1-mCherry PE 12
EMCV-Neo-MYOD1 PE 12
EMCV-Puro-MYOD1-mCherry PE 12
EMCV-Puro-MYOD1 PE 12

Figure S5. Characterization of Tet-MyoD hiPSCs with extended culture, related to Fig 5. **(A)** Representative images of Karyotype for EMCV-Neo-MYOD1-mCherry (middle panel) and EMCV-Puro-MYOD1-mCherry populations (right panel) at PE 3. Healthy control (HC #1) karyotype is shown at left panel. **(B)** qPCR results showing pluripotency markers including *Oct3/4*, *SOX2* and *NANOG* expressions in generated Tet-MyoD hiPSCs at PE 3. Relative expression levels were normalized to *PBGD* as an internal control in each sample and then to HC #1. Values represent means ±SD, n=3. **(C)** Representative images of karyotype for EMCV-Neo-MYOD1-mCherry (middle panel) and EMCV-Puro-MYOD1-mCherry populations (right panel) at PE 12. **(D)** qPCR results showing *Oct3/4*, *SOX2* and *NANOG* expressions in generated Tet-MyoD hiPSCs at PE 12. Relative expression levels were normalized to *PBGD* as an internal control in each sample and then to Healthy control HC #1. Values represent means ±SD, n=3.

Fig S6

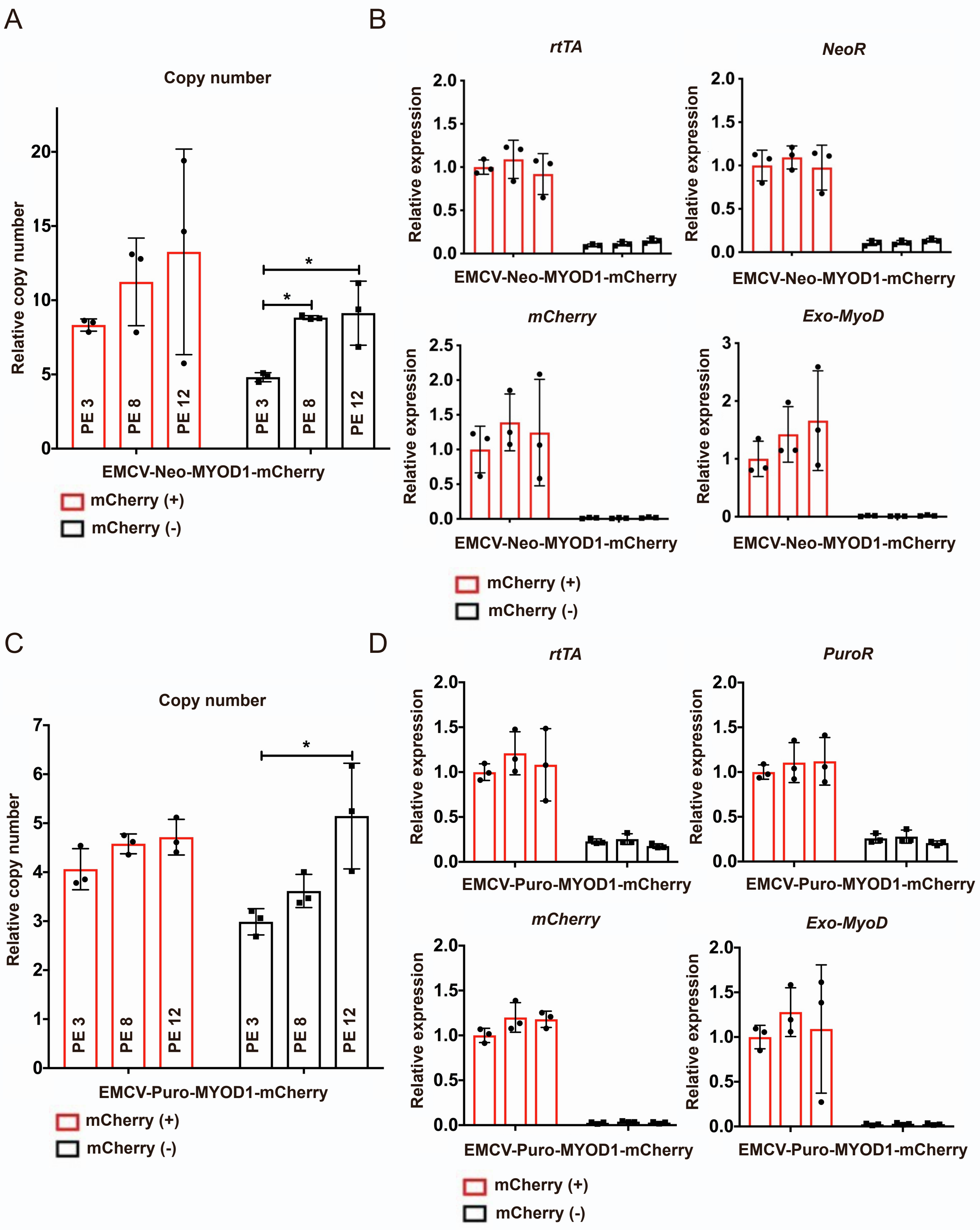
A
Copy number
Relative copy number
PE 3
PE 8
PE 12
PE 3
PE 8
PE 12
*
*
EMCV-Neo-MYOD1-mCherry
mCherry (+)
mCherry (-)
B
rtTA
NeoR
mCherry
Exo-MyoD
Relative expression
EMCV-Neo-MYOD1-mCherry
mCherry (+)
mCherry (-)
C
Copy number
Relative copy number
PE 3
PE 8
PE 12
PE 3
PE 8
PE 12
*
EMCV-Puro-MYOD1-mCherry
mCherry (+)
mCherry (-)
D
rtTA
PuroR
mCherry
Exo-MyoD
Relative expression
EMCV-Puro-MYOD1-mCherry
mCherry (+)
mCherry (-)

Figure S6. Evaluating transgene expression levels and determining copy number integrations in mCherry-positive and -negative populations of Tet-MyoD hiPSCs with extended culture, related to Fig 5 and 6.

(A) qPCR analysis for Tet-MyoD copy number integrations in mCherry-positive and mCherry-negative populations sorted from EMCV-Neo-MYOD1-mCherry at PE 3, PE 8 and PE 12. The rtTA primer was used for detecting Tet-MyoD vectors and DLX5 was used as an internal control. A single copy number integrated Tet-MyoD vector cell line was used as the reference. Values represent means ±SD, n=3. **(B)** qPCR results showing *rtTA, NeoR, mCherry* and *Exo-MyoD* expressions in mCherry-positive and mCherry-negative populations sorted from EMCV-Neo-MYOD1-mCherry at PE 3, PE 8 and PE 12 (from left to right). Relative expression levels were normalized to *PBGD* as an internal control in each sample and then to mCherry-positive populations of EMCV-Neo-MYOD1-mCherry at PE 3. Values represent means ±SD, n=3. **(C)** qPCR analysis for Tet-MyoD copy number integrations in mCherry-positive and mCherry-negative populations sorted from EMCV-Puro-MYOD1-mCherry at PE 3, PE 8 and PE 12. Values represent means ±SD, n=3. **(D)** qPCR results showing *rtTA, PuroR, mCherry* and *Exo-MyoD* expressions in mCherry-positive and mCherry-negative populations sorted from EMCV-Puro-MYOD1-mCherry at PE 3, PE 8 and PE 12 (from left to right). Relative expression levels were normalized to *PBGD* as an internal control in each sample and then to mCherry-positive populations of EMCV-Puro-MYOD1-mCherry at PE 3. Values represent means ±SD, n=3. $^*P<0.05$ according to one-way ANOVA followed by Dunnett's test.

Fig S7

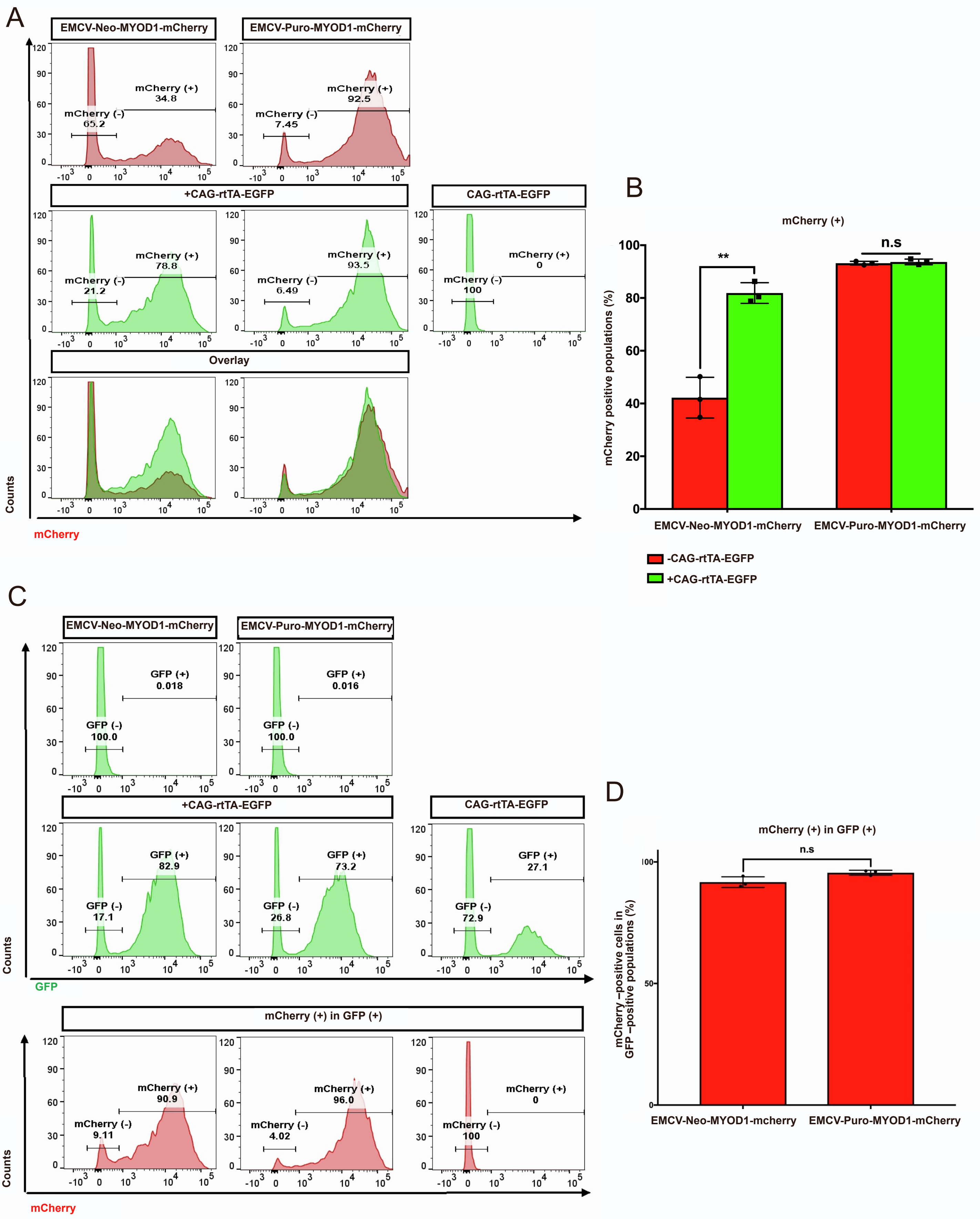
A
EMCV-Neo-MYOD1-mCherry
EMCV-Puro-MYOD1-mCherry
mCherry (+) 34.8
mCherry (-) 65.2
mCherry (+) 92.5
mCherry (-) 7.45
+CAG-rtTA-EGFP
CAG-rtTA-EGFP
mCherry (+) 78.8
mCherry (-) 21.2
mCherry (+) 93.5
mCherry (-) 6.49
mCherry (+) 0
mCherry (-) 100
Overlay
Counts
mCherry
B
mCherry (+)
**
n.s
mCherry positive populations (%)
EMCV-Neo-MYOD1-mCherry
EMCV-Puro-MYOD1-mCherry
-CAG-rtTA-EGFP
+CAG-rtTA-EGFP
C
EMCV-Neo-MYOD1-mCherry
EMCV-Puro-MYOD1-mCherry
GFP (+) 0.018
GFP (-) 100.0
GFP (+) 0.016
GFP (-) 100.0
+CAG-rtTA-EGFP
CAG-rtTA-EGFP
GFP (+) 82.9
GFP (-) 17.1
GFP (+) 73.2
GFP (-) 26.8
GFP (+) 27.1
GFP (-) 72.9
Counts
GFP
mCherry (+) in GFP (+)
mCherry (+) 90.9
mCherry (-) 9.11
mCherry (+) 96.0
mCherry (-) 4.02
mCherry (+) 0
mCherry (-) 100
Counts
mCherry
D
mCherry (+) in GFP (+)
n.s
mCherry –positive cells in GFP –positive populations (%)
EMCV-Neo-MYOD1-mcherry
EMCV-Puro-MYOD1-mCherry

Figure S7. Additional expression of rtTA improves tetracycline-inducible mCherry expression in EMCV-Neo-MYOD1-mCherry hiPSCs, related to Fig 5.

(A) mCherry FCM analysis in EMCV-Neo-MYOD1-mCherry and EMCV-Puro-MYOD1-mCherry populations with (middle row) or without (upper row) CAG-rtTA-EGFP. Bottom row shows overlayed data. Representative graphs are shown. Single electroporation of CAG-rtTA-EGFP served as the negative control. **(B)** Quantification of mCherry-positive populations from the FCM data in Fig S7A. Values represent means ± SD, n=3. **(C)** GFP FCM analysis in EMCV-Neo-MYOD1-mCherry and EMCV-Puro-MYOD1-mCherry populations with (middle row) or without (upper row) CAG-rtTA-EGFP. The mCherry FCM analysis of GFP-positive populations in the same cells is shown at bottom row. Representative graphs are shown. **(D)** Quantification of the mCherry-positive percentage in the GFP-positive populations from Fig S7C. Values represent means ± SD, n=3. **P<0.01 according to Student's *t*-test (unpaired).

Fig S8

A

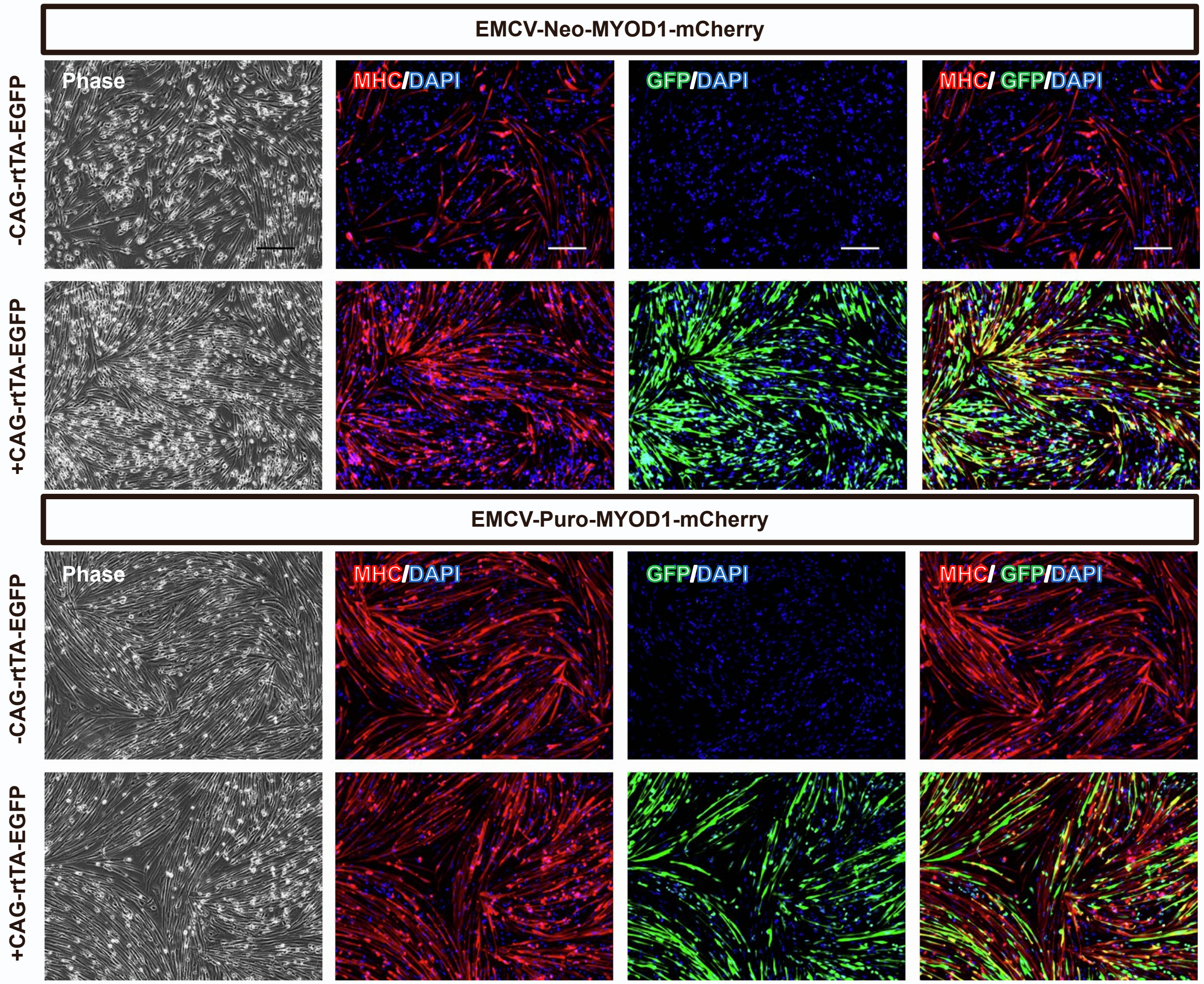

Figure S8. Additional expression of rtTA improves myogenic differentiation in EMCV-Neo-MYOD1-mCherry hiPSCs, related to Fig 5.

(A) Immunostaining for the skeletal muscle cell marker MHC (red) and GFP fluorescence (green) images. DAPI (blue) was used for counter-staining. Differentiated cells were fixed and stained on day7 of the differentiation. Scale bars, 200 µm.

Table S1 The condition of electroporation by NEPA21, related to STAR methods.

	Voltage (V)	Pulse Length (msec)	Pulse Interval (msec)	Number of Pulses	Decay Rate (%)	Polarity
**Poring Pulse**	125.0	5.0	50.0	2.0	10.0	+
**Transfer Pulse**	20.0	50.0	50.0	5.0	40.0	+/−

Table S2 List of antibodies used, related to Figures 1, 2, 5, S1 and S8.

Primary Antibody	Source	Clonarity	Dilution	Company	Catalog Number	RRID
Anti-myosin Heavy chain (MHC) MF20	Mouse monoclonal	IgG2b	1:800	eBioscience	Cat# 14-6503-80	AB_2572893
Anti-MyoD	Rabbit monoclonal	IgG	1:500	Abcam	Cat# ab126726	AB_11130410
Anti-GFP	Chicken polyclonal	IgY	1:500	Thermo Fisher Scientific	Cat# A10262	AB_2534023
Anti-mCherry	Rabbit polyclonal	IgG	1:500	Novus Biological	Cat# NBP2-25157	AB_2753204

Secondary Antibody	Dilution	Company	Catalog Number	RRID
Alexa Fluor 488 goat anti-mouse IgG (H+L)	1:500	Thermo Fisher Scientific	Cat# A-11029	AB_2534088
Alexa Fluor 647 donkey anti-mouse IgG (H+L)	1:500	Thermo Fisher Scientific	Cat# A-31571	AB_162542
Alexa Fluor 647 goat anti-rabbit IgG (H+L)	1:500	Thermo Fisher Scientific	Cat# A-21245	AB_2535813
Alexa Fluor 488 donkey anti-chicken IgY (H+L)	1:500	Thermo Fisher Scientific	Cat# A78948	AB_2921070
Alexa Fluor 568 goat anti-rabbit IgG (H+L)	1:500	Thermo Fisher Scientific	Cat# A-11036	AB_10563566

Table S3 List of Primers for RT-qPCR, related to Figures 1, 3, 4, 5, 6, S1, S3, S4, S5 and S6.

RT-qPCR Primer Sets		
Gene	Forward sequence	Reverse sequence
rtTA	CTGGGAGTTGAAGCAGCCTAC	GAGAGCACAGCGGAATGACT
DLX5	TTCCAAGCTCCGTTCCAGAC	CCCCGTAGGGCTGTAGTAGT
mCherry	CATCCCCGACTACTTGAAGC	CCCATGGTCTTCTGCAT
Exo-MyoD	CCCCTTCACCATGGAGCTA	AGTGCTCTTCGGGTTTCAGG
PBGD	ATTACCCCGGGAGACTGAAC	GGCTGTTGCTTGGACTTCTC
PuroR	CCGAGTACAAGCCCACGG	AGAGTTCTTGCAGCTCGGTG
NeoR	CAAGATGGATTGCACGCAGG	GCAGCCGATTGTCTGTTGTG
Oct3/4	GACAGGGGGAGGGGAGGAGCTAGG	CTTCCCTCCAACCAGTTGCCCCAAAC
Sox2	GGGAAATGGGAGGGGTGCAAAAGAGG	TTGCGTGAGTGTGGATGGGATTGGTG
Nanog	CAGCCCCGA TTCTTCCACCAGTCCC	CGGAAGATTCCCAGTCGGGTTCACC
